# Photodynamically Sensitized Mitochondrial-Damage Amplification Strategy Promotes Pyroptosis-Induced Immunotherapy of Osteosarcoma

**DOI:** 10.34133/bmr.0392

**Published:** 2026-09-09

**Authors:** Tao Ding, Tongyin Xiong, Xinzhong Xu, Qiuting Sun, Mengyu Liu, Zhennan Fang, Yang Li, Zhiwei Wu, Wenbiao Zhang, Ping Zhou, Hongyu Bao, Xiaoyan He, Tao Wang, Chi Zhang, Dasheng Tian, Fei Huang

**Affiliations:** ^1^ Department of Orthopedics, The Second Affiliated Hospital of Anhui Medical University, Hefei 230601, P. R. China.; ^2^ Institute of Orthopaedics, Research Center for Translational Medicine, The Second Affiliated Hospital of Anhui Medical University, Hefei 230601, P. R. China.; ^3^ Biomaterials Research Center, School of Biomedical Engineering, Guangdong Provincial Key Laboratory of Medical Image Processing, Southern Medical University, Guangzhou 510515, China.; ^4^ School of Life Sciences, Anhui Medical University, Hefei 230011, P. R. China.; ^5^ Department of Orthopaedics, Anhui Public Health Clinical Center, Hefei 230000, P. R. China.

## Abstract

The heterogeneity of osteosarcoma (OS) is the main reason for ineffective treatment and uncontrolled disease. Immunotherapy provides an ideal strategy to overcome the heterogeneity of OS, but effective activation of tumor-intrinsic immunogenicity remains challenging. We developed a biomimetic nanoplatform utilizing mitochondrial damage as a potent immunogenic trigger by employing the mitochondria-targeting compound cinnamaldehyde (CA) derived from traditional Chinese medicine. OS cell membrane-coated hollow MnO_2_ nanoparticles were used to deliver CA and chlorin e6. After accumulating at the tumor site and being internalized by tumor cells, CA release induces mitochondrial oxidative damage and produces hydrogen peroxide in combination with hollow manganese dioxide to alleviate the hypoxic microenvironment. Finally, a mitochondrial-damage amplification mechanism is formed to trigger tumor pyroptosis under the synergy of adjustable photodynamic therapy. Released damage-associated molecule patterns and damaged double-stranded DNA inside tumor cells promote dendritic cell maturation by activating the cGAS-STING (cyclic guanosine monophosphate-adenosine monophosphate synthase–stimulator of interferon genes) signaling axis. Through CA-triggered amplification of mitochondrial damage, dual immunogenic pathways reshape the immunosuppressive microenvironment, overcoming OS heterogeneity and inhibiting distal metastasis. This strategy provides new opportunities for tumor-intrinsic immunogenic activation.

## Introduction

Osteosarcoma (OS), the most common primary malignant bone tumor in adolescents, exhibits extreme genetic and spatial heterogeneity that drives metastasis and chemoresistance [1–3]. Despite aggressive multimodal therapy, metastatic OS maintains a 5-year survival below 21.5% [4]. Although immunotherapy offers a promising avenue to overcome heterogeneity by engaging systemic immunity, clinical trials of programmed cell death protein 1/cytotoxic T-lymphocyte-associated protein 4 inhibitors show response rates about 20% [5,6]. This failure stems from the immunosuppressive tumor microenvironment (TME) characterized by hypoxia [7,8], poor immunogenic cell death (ICD), and deficient dendritic cell (DC) activation [9,10]. Critically, a fundamental barrier persists: how to initiate potent tumor-intrinsic immunogenicity that bypasses spatial heterogeneity and triggers systemic antitumor immunity.

Mitochondrial damage is the master switch of immunogenic signaling [11,12]. As the energy factory of tumor cells, the damage and rupture of mitochondria will promote the pyroptosis of tumor cell, damage-associated molecular patterns (DAMPs), and intracytoplasmic-damage double-stranded DNA (dsDNA) are efficiently released under the action of pore-forming in the gasdermin-E [13,14], and the released substances activate DCs through Toll-like receptor 9 and cyclic guanosine monophosphate-adenosine monophosphate synthase–stimulator of interferon genes (cGAS-STING) pathways [15], leading to the formation of new DNA molecules, improving tumor immune microenvironment. Although drugs targeting mitochondria can induce ICD, its clinical translation is still limited by multiple factors [16,17]. The treatment including a single drug is easy to cause nonspecific accumulation of healthy tissues and cause off-target toxicity and cannot achieve cumulative damage beyond the cellular repair threshold. Furthermore, single-agent therapy cannot couple injury to immune adjuvant signaling to overcome TME immunosuppression. Thus, an ideal strategy requires simultaneous activation of innate immune sensors while amplifying mitochondrial damage locally in the tumor [18].

As a bioactive compound in cinnamon, cinnamaldehyde (CA) exhibits a unique mitochondrial tropism by covalently modifying the thiol group in the electron transport chain complex [19–21]. This direct interaction-induced burst of reactive oxygen species (ROS) differs from artificial delivery systems, giving CA extremely high pharmacodynamic potential. However, the intratumoral self-repair mechanism inhibits the effect of CA monotherapy [22]. Therefore, we developed a mitochondrial-damage amplification strategy. First, CA induces mitochondrial stress, which leads to mitochondrial damage and increases hydrogen peroxide production. This process triggers nanocarrier decomposition and remodels the TME. Photodynamic therapy (PDT) further enhances CA-mediated mitochondrial collapse through a tunable approach. Together, these effects establish a positive feedback loop that amplifies mitochondrial damage.

In this study, a biomimetic nanomedicine with hierarchically targeted mitochondrial-damage cycle amplification mechanism was constructed for the first time. Simply put, we coated OS cell membranes on the surface of hollow manganese dioxide (HMnO_2_) loaded with CA and chlorin e6 (Ce6) for safe and targeted delivery of CA and Ce6 accumulation at the tumor site, and we demonstrated that the accumulation of CA and Ce6 at the tumor site is associated with the development of OS cells, avoiding off-target toxicity. Meanwhile, the mitochondrial tropism of CA within tumors facilitates efficient mitochondrial damage and enhances hydrogen peroxide generation. Finally, under the coordinated synergistic effect of tunable PDT-producing ROS storm, a novel approach for the treatment of cancer is proposed, resulting in further impairment of mitochondrial function and membrane integrity triggering pyroptosis. The release of DAMPs and dsDNA through the membrane pore, enhanced by the sensitization of Mn^2+^, greatly increases the activation of the cGAS-STING signaling axis in the peritumoral DC, promoting the maturation of immune cells and remodeling of the tumor immunosuppressive microenvironment. In vitro and in vivo experiments, as well as transcriptomic evidence, support this theory.

Collectively, this controllable damage amplification mechanism based on hierarchically targeting mitochondria shows good potential in triggering OS pyroptosis to improve OS heterogeneity and immune remodeling, providing unique insights for precision immunotherapy of OS.

## Materials and Methods

### Synthesis of HMnO_2_/CA/Ce6@CM (MAC@CM)

We referenced the steps reported in previous literature for the synthesis of hollow mesoporous MnO_2_ nanoshells (HMnO_2_). First, solid silica nanoparticles (sSiO_2_) were synthesized following the reported method. Next, using a 1-pot method, SiO_2_ (1 g) and KMnO_4_ (300 mg) were mixed in a certain ratio and placed in a reaction kettle at 170 °C for 1,500 min; the reactants were washed 3 times with water and alcohol and dried. The dry material was redissolved in water under ultrasonic conditions to obtain MnO_2_-coated sSiO_2_ and then dissolved in 20% NaOH aqueous solution at 50 °C for 24 h. The HMnO_2_ nanoparticles were centrifuged and rinsed with deionized water. Five milliliters of HMnO_2_ solution (2 mg/ml) was mixed with 10 ml of polyallylamine hydrochloride (PAH) solution (5 mg/ml) under constant ultrasonication for 2 h, then centrifuged, and washed thoroughly with water. The content of N atoms in the total sample was calculated by the x-ray photoelectron spectroscopy method, and the loading rate of PAH was indirectly determined. The obtained HMnO_2_/PAH was dispersed in 10 ml of ethanol solution, the pH was adjusted to 8 using 10% sodium hydroxide, and 30 mg of CA was added dropwise under ultrasonication. The reaction temperature was adjusted to 40 °C, and after 5 h, the above solution was centrifuged and washed with ethanol and water to get HMnO_2_/CA. To prepare Ce6-loaded nanoparticles, an HMnO_2_/CA dispersion (HMnO_2_ concentration: 0.2 mg/ml) was incubated with varying concentrations of Ce6 for 12 h. After this incubation, Ce6 was successfully encapsulated into the HMnO_2_ carrier, resulting in the final product designated as HMnO_2_/CA/Ce6. HMnO_2_/CA/Ce6 (HMnO_2_, 0.2 mg/ml) was mixed with the K7M2 cell membrane (collected from 2 × 10^9^ cells) and subjected to ice-bath ultrasonication for 3 h to obtain HMnO_2_/CA/Ce6@CM (MAC@CM, where M is for HMnO_2_, A is for CA, C is for Ce6, and CM is for cell membrane), and the product was used for further experiments.

### In vitro release of CA and Ce6

The dispersions (1 ml) of MA@CM, MC@CM, or MAC@CM were placed in a dialysis bag (molecular weight cutoff of 3,500) and immersed in 9 ml of phosphate-buffered saline (PBS) solution (pH 5.5 or 7.4), intermittently irradiated or unirradiated by the 660-nm laser (1.20 W/cm^2^, 5 min) was given at the time point of 0 h. The samples were incubated in a 37 °C shaking water bath. At designated time points, 1-ml aliquots of the release medium were collected and immediately replenished with an equal volume of fresh medium. The concentrations of released Ce6 and CA in the collected aliquots were separately quantified by ultraviolet–visible (UV–vis) spectroscopy.

### In vitro photodynamic properties of MAC@CM

To evaluate the photodynamic performance of MAC@CM, using 1,3-diphenylisobenzofuran (DPBF) as an indicator, 1-ml solution containing free Ce6 or MAC@CM with various Ce6 concentrations (0.25, 0.5, 1, and 2 μg/ml) was prepared and irradiated with a 660-nm laser (1.20 W/cm^2^, 1 min), and then the MAC@CM was irradiated (containing Ce6 1 μg/ml) for different times (0 min, 1 min, 3 min, and 5 min). The ^1^O_2_ generation capability curve was detected using a fluorescence spectrometer.

### Cells and culture conditions

K7M2 cells were obtained from the China Center for Typical Culture Collection (Wuhan, China). The cells were cultured in Dulbecco’s modified Eagle’s medium supplemented with 10% fetal bovine serum and 1% penicillin–streptomycin at 37 °C in a humidified 5% CO_2_ atmosphere. When the cells reached approximately 80% confluence, they were passaged by digestion with 0.25% trypsin–EDTA. The digestion was terminated by adding complete Dulbecco’s modified Eagle’s medium, and the cell suspension was centrifuged at 1,000 rpm for 5 min. The collected cells were then resuspended in fresh complete medium and subcultured at a split ratio of 1:2.

### In vitro cellular uptake

K7M2 cells were seeded into 35-mm glass-bottom dishes (MatTek) at a density of 10^5^ cells per dish in 1 ml of culture medium and adhered for 24 h. The medium was replaced with 1 ml of fresh medium with test agents (free Ce6, MC@CM, or MAC@CM standardized to 1.6 μM Ce6). After treatment periods of 0, 3, 6, 12, and 18 h, cells were washed 3 times in PBS. Cell nuclei were stained with Hoechst 33342 and visualized with a Zeiss LSM800 confocal laser scanning microscope. Cells were rinsed and harvested, and flow cytometry (FCM) was performed to measure uptake.

### In vivo biodistribution imaging of MAC@CM

For the in vivo study, we used 6-week-old female Balb/c mice. The tumor model was obtained by subcutaneously inoculating 5 × 10^6^ K7M2 cells in the right flank of each mouse. The tumor size reached 150 to 200 mm^3^. Mice were randomly assigned and given either free Ce6 or MAC@CM via tail vein injection (the doses of Ce6 and CA were administered to 4.5 and 5.2 mg/kg). Ce6 biodistribution was monitored noninvasively 3, 6, 12, 24, and 48 h after injection with the PerkinElmer IVIS Lumina III live-imaging system. At the end of 48 h, mice were euthanized, and major organs (heart, liver, spleen, lung, and kidney) and tumors were harvested for ex vivo fluorescence imaging analysis.

### Intracellular ROS generation detection

K7M2 (1 × 10^5^) were seeded in 12-well plates and cultured at 37 °C for 24 h. Then, the cells were supplemented with 1 ml of fresh medium containing the specified agents (M@CM, MA@CM, MC@CM, and MAC@CM; the concentrations of Ce6 and CA in the solutions were 1 and 1.7 μg/ml). Laser irradiation (660 nm, 1.20 W/cm^2^, 5 min) was administered to the relevant groups (Control + L, M@CM + L, MA@CM + L, MC@CM + L, and MAC@CM + L) after they had been coincubated at 37 °C for 12 h. After incubation for another 4 h, the cells were stained with 2′,7′-dichlorofluorescin diacetate-containing medium (10 μM) for another 25 min. Finally, the intracellular fluorescence of ROS (λex = 488 nm, λem = 528 nm) was detected using a microplate reader (Mshot, MF52-N) and FCM.

### Live/dead cell staining analysis

For the live/dead cell staining analysis, K7M2 cells (1 × 10^5^) were plated and cultivated in 12-well plates for 24 h. Then, the culture medium was replaced with 1 ml of fresh medium containing the particular agents (M@CM, MA@CM, MC@CM, and MAC@CM; the concentrations of Ce6 and CA in the solutions were 1 and 1.7 μg/ml). Subsequently, after 12 h of coincubation at 37 °C, the cells were subjected to laser irradiation at 660 nm (1.20 W/cm^2^, 5 min) in the groups of Control + L, M@CM + L, MA@CM + L, MC@CM + L, and MAC@CM + L. After incubation for another 4 h, a dual stain with calcein-AM and propidium iodide was applied for 20 min. Cellular fluorescence, indicative of viability status, was subsequently visualized and imaged with an inverted fluorescence microscope (Mshot, MF52-N).

### In vitro mitochondrial-damage assays

K7M2 cells were prepared as a suspension at a density of 1 × 10^5^ cells in 1 ml of culture medium. This suspension was then plated into a 35-mm glass-bottom culture dish. After incubation for 24 h, the culture medium was replaced with 1 ml of fresh medium containing the particular agents (M@CM, MA@CM, MC@CM, and MAC@CM; the concentrations of Ce6 and CA in the solutions were 1 and 1.7 μg/ml). After coincubation at 37 °C for 12 h, the laser irradiation (660 nm, 1.20 W/cm^2^, 5 min) was performed in the groups of Control + L, M@CM + L, MA@CM + L, MC@CM + L, and MAC@CM + L. After incubating for another 4 h, the cells were stained by the JC-1 working solution and observed with confocal laser scanning microscopy (CLSM).

### Apoptosis analysis

K7M2 cells (1 × 10^5^) were plated and cultivated in 12-well plates for 24 h. Then, the culture medium was replaced with 1 ml of fresh medium containing the particular agents (M@CM, MA@CM, MC@CM, and MAC@CM; the concentrations of Ce6 and CA in the solutions were 1 and 1.7 μg/ml). Following a 12-h coincubation period at 37 °C, the designated experimental groups (Control + L, M@CM + L, MA@CM + L, MC@CM + L, and MAC@CM + L) were subjected to laser irradiation (660 nm, 1.20 W/cm^2^, 5 min). After an additional 4 h of incubation, cell apoptosis was analyzed. This was performed by staining the cells with an Annexin V-fluorescein isothiocyanate/propidium iodide apoptosis detection kit according to the manufacturer’s instructions, followed by quantitative assessment using FCM.

### In vitro bone marrow-derived DC extraction and activation

Bone marrow-derived DCs were isolated under sterile conditions from the femurs and tibias of 6-week-old female BALB/c mice. The bone marrow cell suspension was first filtered through a 100-μm cell strainer to remove tissue debris. Cells were pelleted by centrifugation at 2,000 rpm for 5 min. To removed red blood cells, the pellet was treated with an erythrocyte lysis buffer for 10 min at room temperature. After 2 washes with PBS and centrifugation, the resulting cell pellet was collected. The purified cells were then resuspended in complete RPMI 1640 medium supplemented with 10% fetal bovine serum, 1% penicillin–streptomycin, 20 ng/ml recombinant murine granulocyte-macrophage colony-stimulating factor, and 20 ng/ml interleukin-4. Cells were seeded into 6-well plates at a density of 2 × 10^5^ cells per well for differentiation. Following a 7-d culture period, the medium was changed to a conditioned medium collected from pretreated K7M2 cells. After a further 24-h coculture, both DCs and K7M2 cells were harvested and subjected to staining with fluorochrome-conjugated antibodies for subsequent analysis by FCM (i.e., APC/Fire 750 anti-mouse CD45, fluorescein isothiocyanate anti-mouse CD11c, P-phycoerythrin anti-mouse CD80, and Brilliant Violet 421 anti-mouse CD86).

### In vivo antitumor efficacy

All animal experiments were carried out in accordance with the relevant laws and guidelines of the Laboratory Animal Center of Anhui Medical University (approved number: LLSC 20200329). To generate a primary mouse model bearing K7M2 tumors, female Balb/c mice were inoculated subcutaneously in the right flank with K7M2 cells (5 × 10^6^ cells/mouse). After 1 week, tumor-bearing mice were randomly assigned to 5 experimental groups (5 mice per group). Mice in each group subsequently received 2 rounds of intravenous treatment with one of the following: M@CM, MA@CM, MC@CM, or MAC@CM. The dosage was standardized at 4.5 mg/kg for Ce6 and 5.2 mg/kg for CA per administration. Twelve hours after every administration, the mice were irradiated with light (660-nm laser, 1.20 W/cm^2^, 5 min). Throughout the treatment period, tumor volume and body weight were recorded at 2-d intervals. Tumor volume was calculated using the formula: Volume (mm^3^) = (width^2^ × length) × 0.5. The tumor suppression rate was calculated as the relative tumor volume (RTV = *V*/*V*_0_), where *V*_0_ and *V* denote initial and posttreatment tumor volumes, respectively. Tumor suppression rate was defined as (RTVc-RTVx)/RTVc, subscripts c and x representing the control group and the treatment group. Day 14, after measurement, all mice were euthanized. Major organs (heart, liver, spleen, lung, and kidney) and tumor tissues were harvested. The tissues were fixed in 4% paraformaldehyde, subsequently embedded in paraffin, sectioned into 5-μm slices, and stained with hematoxylin and eosin (H&E) for light microscopy. Tumor cell apoptosis was assessed via TUNEL (terminal deoxynucleotidyl transferase dUTP nick end labeling) immunofluorescence staining. To evaluate ICD, calreticulin (CRT) exposure was examined by immunofluorescence and visualized using CLSM. Additional tumor samples were collected for imaging and immune profiling. The proportions of DCs, regulatory T cells (Tregs), and cytotoxic T lymphocytes (CTLs) in the spleen, lymph nodes, and tumors were determined by FCM. A parallel control group received intravenous injections of PBS and underwent identical analyses.

### Antitumor effect of bilateral tumor model

To establish primary and distal tumor models, K7M2 cells (5 × 10^6^ cells per mouse) were subcutaneously inoculated on the right and left sides of female Balb/c mice, respectively, before the start of treatment. When the primary tumor volume reached about 150 mm^3^, the tumor-bearing mice were randomly divided into 2 groups (*n* = 5 each). Each group received 2 rounds of intravenous treatment with PBS or MAC@CM, with the latter administered at doses of Ce6 4.5 mg/kg and CA 5.2 mg/kg per mouse. After each injection, 24 h later, the primary tumor was laser irradiated (660 nm, 1.20 W/cm^2^, 5 min). Volumes and body weights of primary and distant tumors were recorded every 2 d for 14 d. At the end point (day 14), all mice were euthanized, and tumor tissues were collected for photographic recording and subsequent immunoassay. The proportion of DCs, Tregs, and CTLs in spleen, lymph nodes, primary tumors, and distant tumors was determined by FCM.

### Statistical analysis

All experiments were repeated at least 3 times to obtain definitive results. Data are presented as means ± SD. Sample sizes for in vitro and in vivo analyses are annotated in the figure legends. Student *t* test (2-tailed) and 1-way analysis of variance (ANOVA) (Tukey’s multiple comparisons test) were used for statistical analysis. Data were collected using GraphPad PRISM 9.5.1 software, and fluorescence data were analyzed using ImageJ software.

## Results and Discussion

### Preparation and characterization of the MAC@CM

**Fig. 1.**
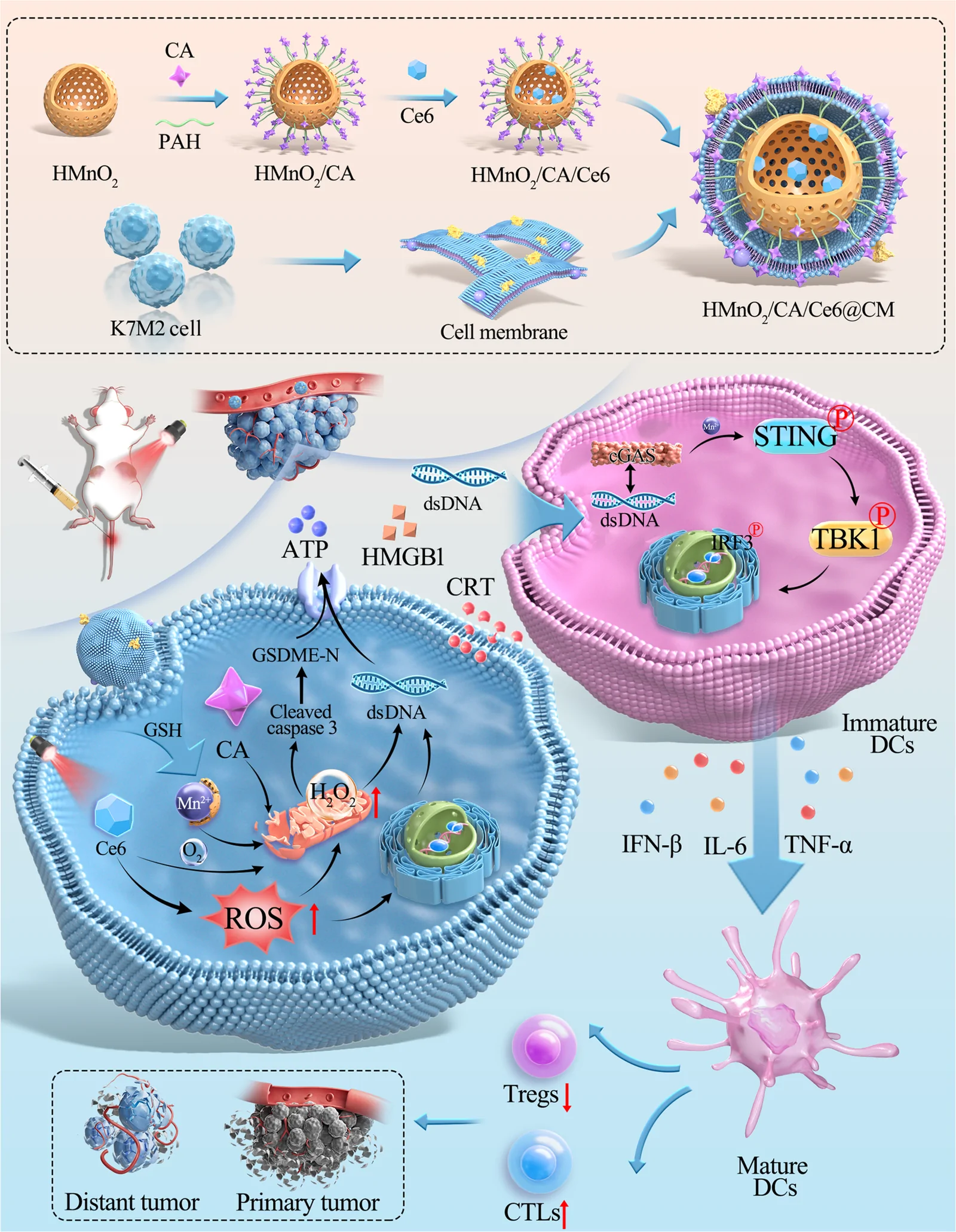
Synthetic concept of MAC@CM and the dual immunopotentiating effect of MAC@CM via pyroptosis-immunogenic cell death and activation of the cyclic guanosine monophosphate-adenosine monophosphate synthase–stimulator of interferon genes (cGAS-STING) pathway. IFN-β, interferon-β; IL-6, interleukin-6; TNF-α, tumor necrosis factor-α; TBK-1, TANK-binding kinase 1; IRF-3, interferon regulatory factor 3.

**Fig. 2.**
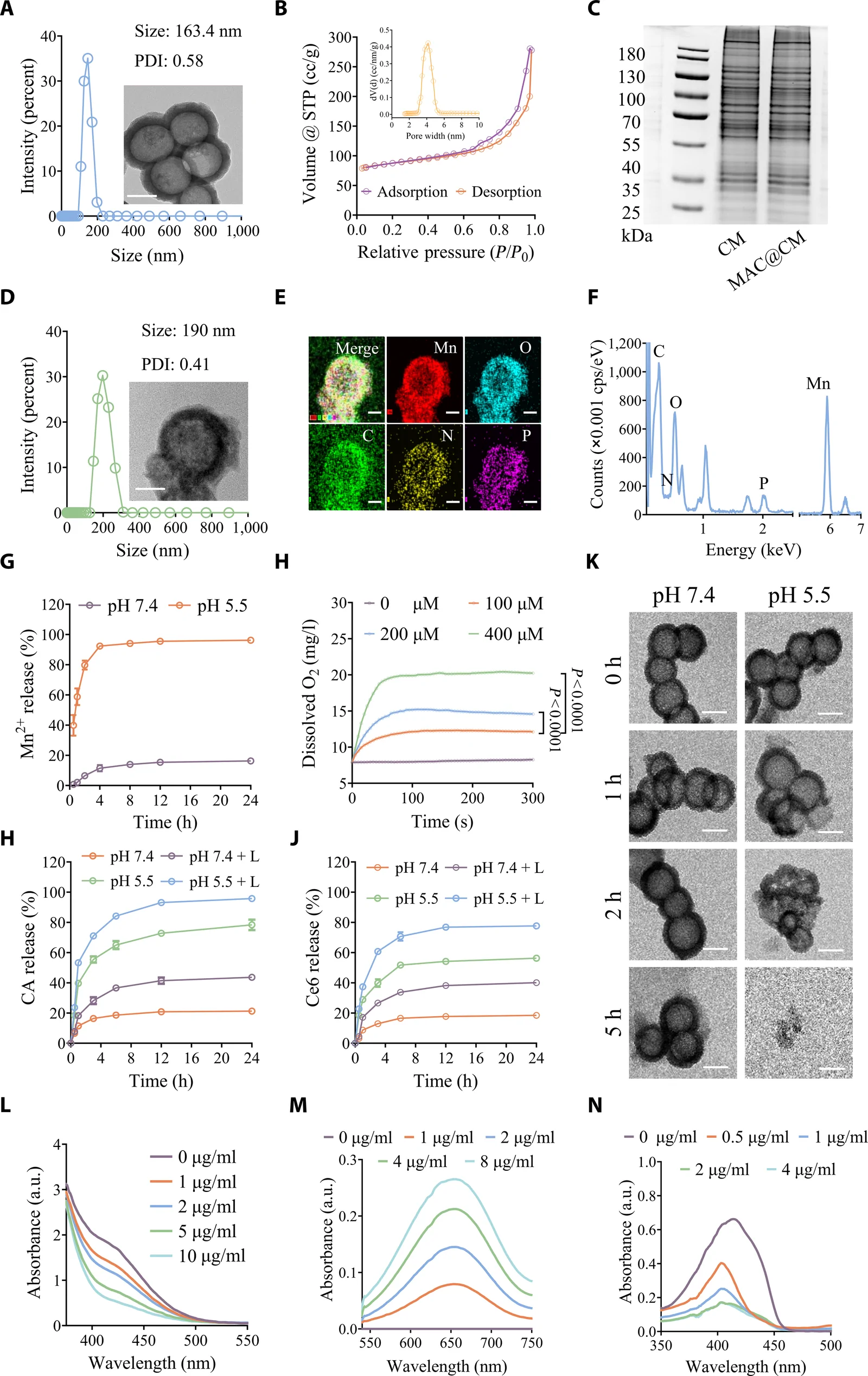
Synthesis and characterization of MAC@CM. (A) The dynamic laser scattering (DLS) analysis and transmission electron microscope (TEM) image of hollow manganese dioxide (HMnO_2_). Scale bar: 50 nm. (B) Brunner–Emmet–Teller analysis of HMnO_2_ for drug-carrying capacity. (C) Protein profiles of CM and MAC@CM. (D) The DLS analysis and TEM image of MAC@CM. Scale bar: 50 nm. (E) The high-angle annular dark-field scanning TEM and (F) energy dispersive x-ray spectroscopy energy spectrum analysis for MAC@CM. (G) Inductively coupled plasma analysis for MAC@CM. (H) The detection for oxygen production capacity of MAC@CM. The drug release of (I) cinnamaldehyde (CA) and (J) chlorin e6 (Ce6) in MAC@CM. (K) TEM image analysis for MnO_2_ degradation behavior. Scale bar: 50 nm. (L) Glutathione (GSH) consumption by different MAC@CM. The chemodynamic therapy (CDT) and singlet oxygen analysis by (M) ultraviolet–visible curve of 3,3ʹ,5,5ʹ-tetramethylbenzidine and (N) 1,3-diphenylisobenzofuran. The laser (660 nm) power density and irradiation time were 1.20 W/cm^2^ and 1 min, respectively. Data in (G), (I), and (J) are reported as the mean values ± SD, *n* = 3.

Figure 1 describes the synthesis of MAC@CM. First, silica nanoparticles act as a hard template to produce a uniform manganese dioxide layer from potassium permanganate. HMnO_2_ nanoparticles were synthesized by treating the silica-manganese dioxide intermediate with a sodium carbonate solution. Transmission electron microscope (TEM) analysis revealed that the obtained HMnO_2_ nanoparticles exhibit a spherical and hollow morphology, with an average diameter of approximately 162 nm (Fig. 2A). Next, in order to investigate the drug loading properties of HMnO_2_, Brunauer–Emmett–Teller measurements showed that the specific surface area and average pore size of HMnO_2_ were 359.66 m^2^/g and 4.15 nm, respectively (Fig. 2B). It is expected to be an ideal high efficiency drug carrier material. In order to explore the drug-carrying capacity of HMnO_2_, Ce6 was mixed with HMnO_2_ in an appropriate concentration in an ultrasonic environment and then stirred, and free substances were removed in a light-protected environment to obtain Ce6@HMnO_2_. In order to load CA, we first modified HMnO_2_ surface with PAH, then conjugated CA to the HMnO_2_ surface by Schiff base reaction, and obtained about 51.96% loading ratio (Fig. S1). The loading capacity of Ce6 was determined by the UV–vis absorption spectrum. When the ratio of material to weight (Ce6: HMnO_2_) is 3:1, the loading ratio of Ce6 reaches 84.9%. The full spectrum of UV absorption peaks for each substance was measured, indicating successful drug loading (Fig. S2). Meanwhile, the MAC curve is similar to the free-Ce6 curve, which provides the possibility for MAC to be used in a PDT treatment.

The biocompatibility, solution stability, and tumor targeting ability of tumor cell membrane-encapsulated nanomedicines (integrin β1, integrin αV, N-cadherin, and adhesion plaque kinase) [23,24] are important considerations. Therefore, we encapsulated K7M2 cell membranes onto the surface of manganese dioxide (HMnO_2_) using ultrasound and electrostatic adsorption to form MAC@CM nanoparticles. To investigate successful coating of membranes, the Coomassie Blue method was applied to detect protein retention on MAC@CM (Fig. 2C). The protein profile traced on MAC@CM was similar to CM, indicating that most CM proteins remained on the surface of MAC@CM. At the same time, TEM images of MAC@CM nanoparticles clearly show the spherical appearance of a transparent coating with a diameter of about 25 nm (Fig. 2D). Elemental imaging based on high-angle annular dark-field scanning TEM (Fig. 2E) and energy-dispersive spectroscopy analysis (Fig. 2F) further confirmed the film coating of MAC@CM nanoparticles. Furthermore, the x-ray diffraction patterns of MAC (36.15°, 65.75°) and MAC@CM (36.15°, 65.77°) show similar characteristic peaks, consistent with literature reports [25], indicating that drug loading and surface modification of cell membranes do not affect the crystal structure of nanoparticles (Fig. S3). The hydrodynamic size of HMnO_2_, MA, MC, and MAC@CM was then determined by dynamic laser scattering (Fig. S4). The particle size of MAC@CM increases with the increase in drug loading and modification of membrane surface, which indicated that the particle size of MAC@CM increases after coating. Furthermore, the zeta potential of the CM-modified group decreased to negative values compared to the other groups, further confirming successful modification of the membrane surface (Fig. S4). Overall, these results indicated that CM successfully modifies the HMnO_2_ surface. Stability testing in PBS over a 7-d period revealed that all intermediate products maintained stable particle sizes, with only negligible changes observed (Fig. S5). In addition, we used hemolysis experiments to evaluate the biological safety of MAC@CM at different concentrations, and even the maximum concentration of MAC@CM (200 μg/ml, HMnO_2_) had a hemolysis rate of less than 4%, which ensured the blood safety of MAC@CM in vivo (Fig. S6).

In order to validate MAC@CM as a biomimetic nanomedicine delivery platform for photoimmunity, we conducted corresponding in vitro solution experiments. First, MAC@CM was placed in a dialysis bag and placed in pH 7.4 and pH 5.5 environment, respectively. Inductively coupled plasma atomic emission spectroscopy detection was performed on the solution outside the dialysis bag at different times (Fig. 2G). The results showed that MAC@CM had excellent degradation efficiency in the solution simulating TME and responding to TME, releasing a large amount of Mn^2+^. Considering that oxygen is an important element in the formation of PDT [26,27], the presence of endogenous H_2_O_2_ in the range of 10 to 100 μM in most solid tumors was confirmed. The ability of MAC@CM to catalyze H_2_O_2_ decomposition was tested by adding different concentrations of H_2_O_2_ to the MAC@CM solution, measuring dissolved oxygen by oxygen probe, and detecting H_2_O_2_ in solid tumors. Although the dissolved O_2_ level in H_2_O_2_ solution was stable without MAC@CM addition, we found that MAC@CM could effectively trigger H_2_O_2_ to produce O_2_ rapidly in a H_2_O_2_ concentration-dependent manner (Fig. 2H). Then, we measured the relative cumulative drug release under different environmental conditions (Fig. 2I and J), and the results showed that the TME and light conditions were conducive to drug release. Finally, we examined the degradation behavior of MAC@CM in different environments using TEM (Fig. 2K), which further matched the previous data.

To assess the ability of MAC@CM to deplete glutathione (GSH), a key intracellular antioxidant whose levels substantially affect PDT efficacy, MAC@CM was added to GSH solution at various concentrations. The remaining GSH content was quantified using 5,5ʹ-dithiobis (2-nitrobenzoic acid) as a detection reagent, followed by UV–vis spectroscopy after a 30-min incubation period (Fig. 2L); MAC@CM efficiently consumes GSH in a concentration-dependent manner, providing a favorable intracellular environment for PDT. In addition, we also examined CA components in MAC@CM and found that CA also partially depleted GSH in an acidic environment (Fig. S7A). We then verified the ability of MAC@CM degradation product Mn^2+^ to act as a Fenton reagent, exert chemodynamic therapy efficiency, and generate ROS, using 3,3ʹ,5,5ʹ-tetramethylbenzidine as a chromogenic reagent for hydroxyl radicals and adding different concentrations of MAC@CM to solutions containing 3,3ʹ,5,5ʹ-tetramethylbenzidine. After incubation at 37 °C for 30 min, hydroxyl radical generation was detected by UV–vis spectroscopy (Fig. 2M and Fig. S7B). The results showed that the chemodynamic therapy activity of MAC@CM was concentration-dependent, and more hydroxyl radicals were produced with the increase in concentration. Finally, we used DPBF as an indicator of singlet oxygen (^1^O_2_) to test MAC@CM for PDT capability under conditions conducive to PDT environment formation. Different concentrations of MAC@CM were added to ethanol containing DPBF, irradiated at 1.2 W for 1 min with 660-nm laser light in the dark and immediately detected by UV–vis (Fig. 2N). The results indicated that MAC@CM could effectively enhance the production of ^1^O_2_ in a concentration-dependent manner. Furthermore, the results of illumination at different time points showed that the amount of ^1^O_2_ produced by MAC@CM was time-dependent (Fig. S7C).

### Internalization and biodistribution of nanoplatforms

In cancer therapeutics, nanoparticles coated with tumor cell membranes have attracted substantial interest because of their ability to mimic biological properties. Membrane-coated systems exhibit improved biocompatibility, efficient immune escape, and natural tropism in tumor tissues, which collectively contribute to prolonged circulation and enhanced pharmacokinetics [28]. The homologous targeting mechanism is due to functional proteins, integrins, adhesion kinases, and Rho family proteins, embedded in the phospholipid bilayer of the source cell membrane [23,24,29]. Thus, nanoparticles cloaked with K7M2 cell-derived membranes acquire a precise homing ability toward homologous OS in vivo, improving the delivery efficiency of the therapeutic system.

**Fig. 3.**
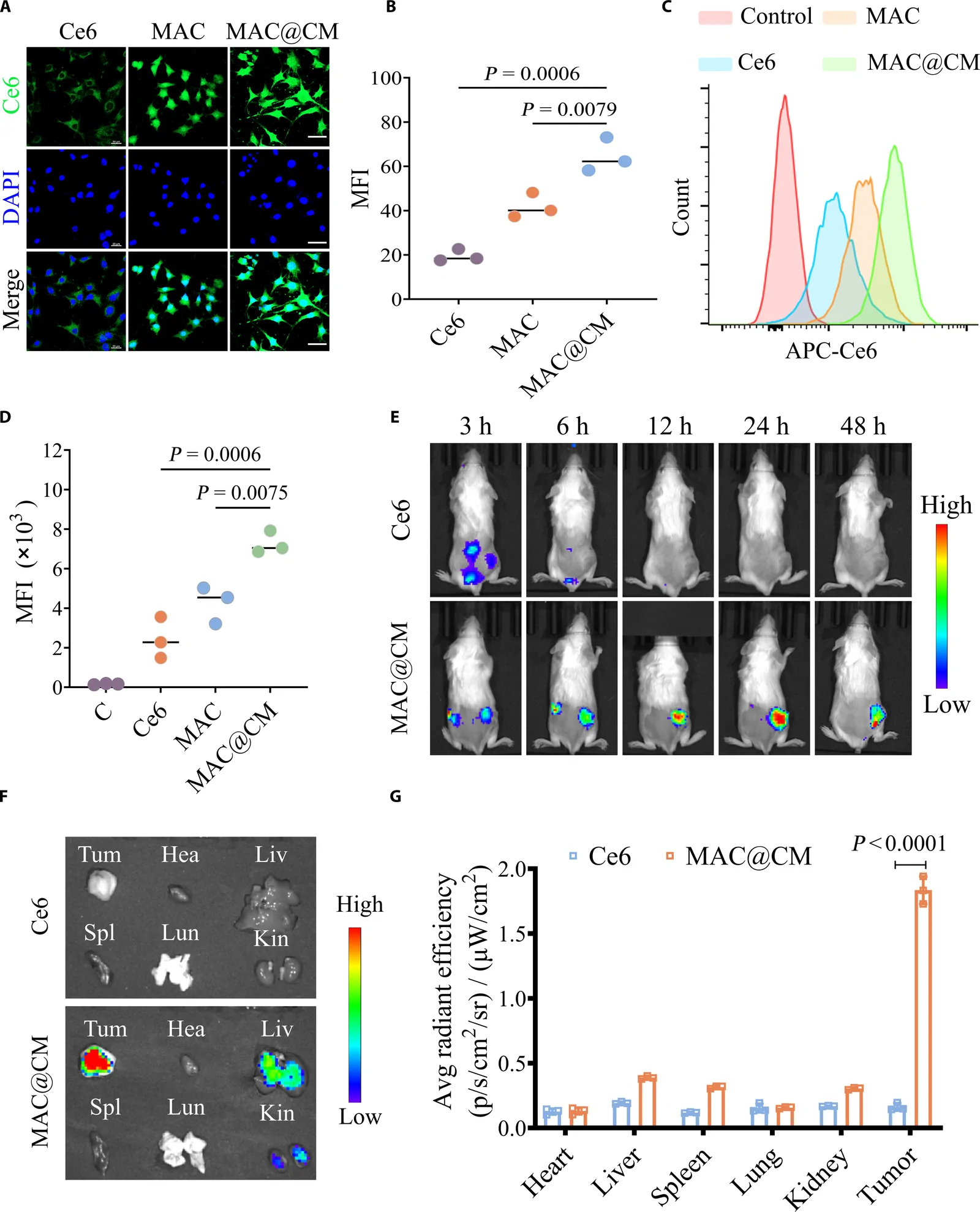
Evaluation of the cellular uptake and tumor cleavage of MAC@CM. (A and B) Confocal laser scanning microscopy observation and (C and D) flow cytometry analysis of the cellular uptake of nanoplatforms. Cells were treated with free chlorin e6 (Ce6), MAC, and MAC@CM for 12 h. Scale bar: 20 μm. Nuclei were labeled with 4′,6-diamidino-2-phenylindole (DAPI) (blue fluorescence). Ce6 was excited to emit green fluorescence. (E) Fluorescence images of K7M2 tumor-bearing mice after injecting with free Ce6, MAC@CM for various periods. Ex vivo (F) fluorescence images and (G) fluorescence intensities of major organs and tumors at 48 h after injection. Data in (B), (D), and (G) are reported as the mean values ± SD, *n* = 3. The statistical significance was obtained using 1-way analysis of variance (ANOVA). MFI, mean fluorescence intensity.

To assess tumor targeting properties of MAC@CM, we measured its drug accumulation in vitro and in vivo experiments. We found that green fluorescent Ce6 carried by MAC@CM colocated with blue nuclei of tumor cells, which confirms efficient uptake. Compared to cells treated with free Ce6 and MAC, the green fluorescence of MAC@CM cells was stronger (Fig. 3A and B), suggesting more drug accumulation. The drug accumulation detected by FCM was consistent with the results of CLSM (Fig. 3C and D). Time-course analysis of MAC@CM endocytosis revealed that accumulation reached saturation 12 h after treatment (Fig. S8). In addition, we examined the assessment of fluorescence colocalization of lysosomes with MAC@CM in K7M2 cells during this time period (Fig. S9), and the results showed a time-dependent aggregation of MAC@CM within lysosomes. We set the initial laser-irradiation time 12 h in cell experiments. Together, these results suggest that membrane coating substantially improves intracellular integration efficiency of the nanoparticle platform and thus strongly supports the therapeutic potential of MAC@CM in vitro applications.

The tumor accumulation of MAC@CM was measured in vivo using a small-animal fluorescence imaging system. After tail vein injection in K7M2 tumor-bearing mice, the real-time biodistribution of nanoplatform was monitored. Fluorescence in tumors region was weak in the free-Ce6 group (Fig. 3E). However, the MAC@CM group was fluorescent in tumor region due to enhanced permeability and retention effect. Fluorescence intensity in tumor tissue was highest 24 h after injection of MAC@CM, and fluorescence could still be observed for 48 h due to the higher concentration and longer retention of MAC@CM in tumor area. Overall, the results showed that MAC@CM allowed tumor targeting and preferential accumulation at the tumor site. According to tumor accumulation kinetics, in vivo laser irradiation began at 24 h after injection, the time point of peak fluorescence intensity. This was followed by in vitro imaging of the tumor and major organs within 48 h. The fluorescence signal was mainly localized within tumor tissues (Fig. 3F and G), indicating that the biomimetic nanoplatform has a distinct tumor-selective distribution. As targeted enrichment of therapeutic agents is crucial for therapeutic efficacy, confirmed accumulation supports further investigation of the antitumor mechanisms and therapeutic outcomes of this platform at the cellular and animal levels.

### Enhanced generation of ROS and pyroptosis activation

Cytotoxicity is key to ensuring successful antitumor effects. Therefore, before validating the antitumor mechanism of MAC@CM, we first conducted a Cell Counting Kit-8 (CCK-8) assay to determine the appropriate lethal drug concentration on K7M2 cells. Based on the previously mentioned drug loading rate, and considering sufficient loading while ensuring minimal material waste, we synthesized the drug in a ratio of HMnO_2_:Ce6:CA = 1:1:2. As shown in Fig. S10A, M@CM, lasered or not, is weakly lethal against K7M2 cells even at 20 μg/ml, and hence, pure HMnO_2_ is not very effective against K7M2. We conducted the same cytotoxicity experiments on MAC@CM. As shown in Fig. S10B, MAC@CM exhibited a dose-dependent lethality against K7M2 cells, and the lethality substantially increased after laser irradiation. Specifically, under laser irradiation, the concentration of Ce6 in MAC@CM reached 1.68 μM, demonstrating a half-maximal inhibitory concentration (IC50). We then performed cell viability assays on MA@CM and MC@CM using the same concentration range. As shown in Fig. S10C, MC@CM exhibited negligible cytotoxicity without laser irradiation. However, laser irradiation produced a certain level of toxicity. The IC50 concentration of Ce6 was 3.35 μM in the MC@CM + L group. The cell viability in the MA@CM group remained largely unchanged with or without laser irradiation (Fig. S10D), and the MAC@CM + L treatment exhibited the lowest cell survival rate at each corresponding drug concentration. Therefore, we used the IC50 of the drug in MAC@CM as a reference, employing drug concentrations of Ce6: 1.68 μM and CA: 2.0 μg/ml for subsequent cell experiments. To further evaluate the tumor-selective safety of MAC@CM, human umbilical vein endothelial cells were treated with different concentrations of different components and analyzed by CCK-8 assay. The results showed negligible cytotoxicity toward normal human umbilical vein endothelial cells at the therapeutic concentration (Fig. S10E), while MAC@CM + L markedly reduced the viability of K7M2 tumor cells, indicating acceptable cytocompatibility and tumor-preferential therapeutic activity. Furthermore, to quantitatively determine whether the combined chemotherapy/PDT treatment was synergistic or additive, the combination index (CI) was calculated using the Chou–Talalay method. The CI values of MAC@CM were markedly lower than 1 across the tested effect levels (Fig. S11). Moreover, the CI values obtained from the actual experimental points ranged from 0.07244 to 0.30455, further confirming a robust synergistic interaction between the chemotherapeutic and PDT components. These results indicate that MAC@CM enhances antitumor efficacy through synergistic chemotherapy/PDT rather than a simple additive effect. Simultaneously, Fig. S10A to D shows that the control group’s effect of illumination is also negligible. To simplify subsequent experimental operations, the experimental groups were set as Control + L, M@CM + L, MA@CM + L, MC@CM + L, and MAC@CM + L.

**Fig. 4.**
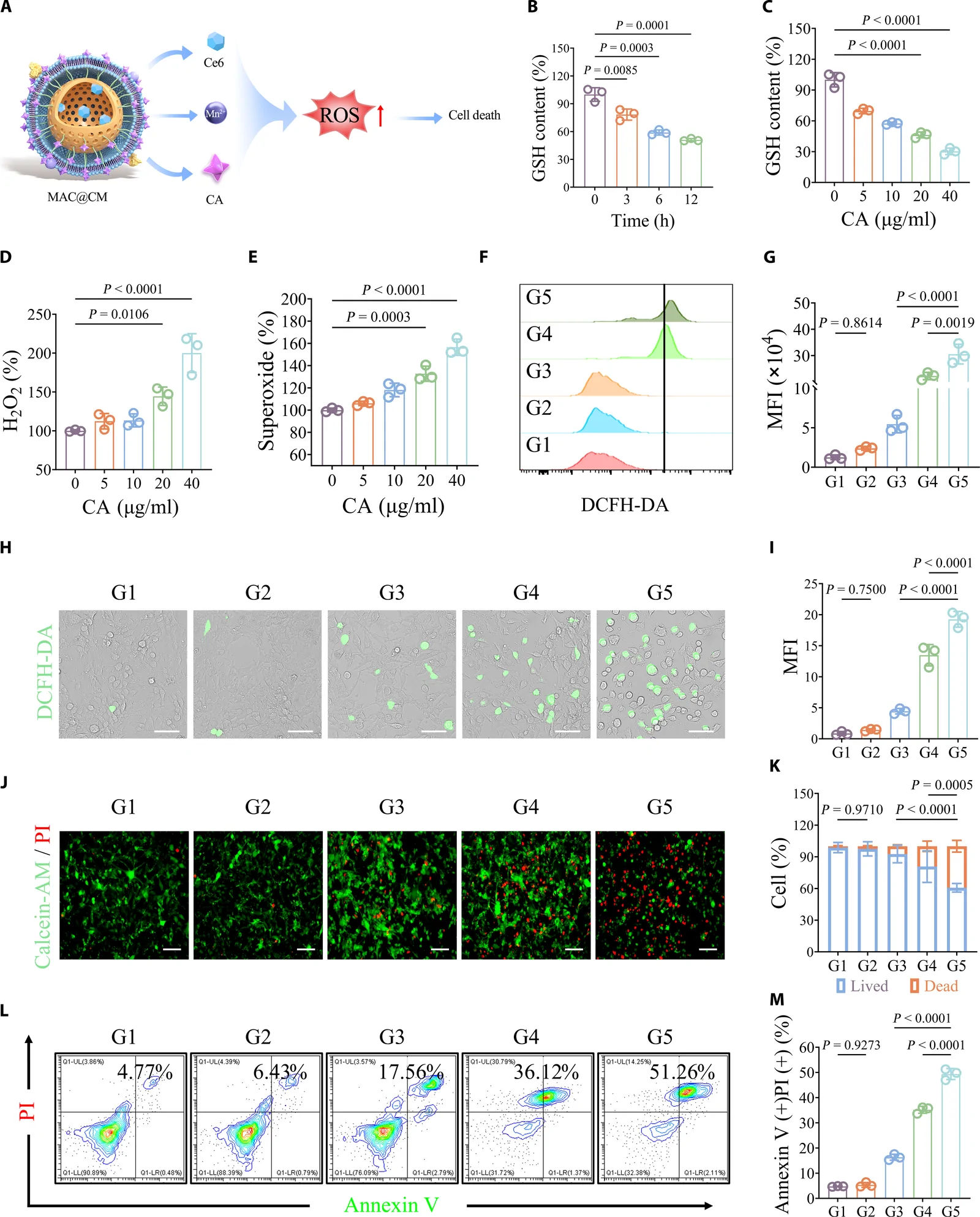
In vitro evaluation on the synergistic antitumor effects of MAC@CM. (A) The mechanism of MAC@CM inducing the cell death under laser irradiation. The microenvironment facilitates the remodeling of photodynamic therapy through (B) time- and (C) concentration-dependent glutathione (GSH) depletion and (D) H_2_O_2_ and (E) superoxide generation. (F and G) Flow cytometry (FCM) analysis and (H and I) microscopic observation of intracellular reactive oxygen species (ROS) levels in K7M2 cells following different treatments. Scale bar: 100 μm. (J and K) Live/dead assay and (L and M) FCM analysis of K7M2 cells after different treatments. Scale bar: 100 μm. The laser (660 nm) power density and irradiation time were 1.20 W/cm^2^ and 5 min, respectively. G1: Control + L, G2: M@CM + L, G3: MA@CM + L, G4: MC@CM + L, and G5: MAC@CM + L. Data in (B to E), (G), (I), (K), and (M) are presented as the mean values ± SD, *n* = 3. The statistical significance was obtained using a 1-way analysis of variance (ANOVA). MFI, mean fluorescence intensity.

As shown in the diagram (Fig. 4A), after the drug enters the cell, HMnO_2_ and CA disrupt the intracellular GSH antioxidant system, reducing the tumor cells’ resistance to subsequent PDT damage. At the same time, based on HMnO_2_ and CA, H_2_O_2_, oxygen, and superoxide are provided, creating a favorable environment for PDT. After laser irradiation, ROS production increases rapidly. To prove this, first we measured GSH content in the cells at 0, 3, 6, and 12 h after MAC@CM. As shown in Fig. 4B and C, we found that GSH decreases time dependently and concentration dependently and decreased to about 50.97% in 12 h. We assessed the ability of different components to deplete GSH (Fig. S12A) and found that HMnO_2_ and CA combine to consume GSH, further enhancing the ability of the nanoplatform to disrupt the oxidative system, laying the foundation for subsequent PDT. Next, we evaluated the ability of the MAC@CM nanoplatform to generate H_2_O_2_, as shown in Fig. 4D, the result showed that MAC@CM has a concentration-dependent ability to generate H_2_O_2_, providing sufficient sources for subsequent oxygen generation. Since HMnO_2_ degradation in the TME produces superoxide, we added different concentrations of MAC@CM to K7M2 cells and measured intracellular superoxide content after 12 h. The results are consistent with Fig. 4D, showing a concentration-dependent formation of superoxide (Fig. 4E). Furthermore, we assessed the ability of different groups to generate hydrogen peroxide and superoxide, finding that MAC@CM has a substantially higher ability to generate both compared to other groups (Fig. S12B and C).

Next, we added MAC@CM to the cells and incubated for 12 h, irradiated with a 660-nm laser, and measured intracellular fluorescence intensity using FCM by 4 h later. As shown in Fig. 4F and G, we found that after laser irradiation, the group containing Ce6 showed a substantial increase in ROS content, with the MAC@CM + L group having the highest content. To characterize ROS content more intuitively, we then examined intracellular ROS fluorescence by CLSM, and the results were similar to those of FCM, indicating that the MAC@CM + L group had substantial ROS generating capacity (Fig. 4H and I). The experimental results confirm that the initial damage of the MAC@CM intracellular antioxidant system by HMnO_2_ and CA creates a favorable environment for the application of PDT. Subsequently, we employed the CCK-8 detection kit to detect the cytotoxicity of MAC@CM on K7M2 cells. As shown in Fig. S12D, among K7M2 cells treated under different conditions, the MAC@CM + L group showed about 72.42% cytotoxicity, which was the strongest among the other groups. Further staining by live/dead cells and microscopy showed agreement with the CCK-8 data (Fig. 4J and K). FCM analysis was consistent with microscopic observations, with the highest cell death rate in the MAC@CM + L group (Fig. 4L and M). In addition, we used healing assay to detect the cell migration of different groups at different times (Fig. S13). The results showed that within 24 h, after laser irradiation, compared with the migration process of the Control group, the cells in the MAC@CM group died and did not migrate. These results indicate that MAC@CM can kill K7M2 cells under laser irradiation, and HMnO_2_ and CA synergistically enhance the cytotoxic effect of Ce6, suggesting that MAC@CM has substantial potential as an effective adjuvant for tumor treatment.

**Fig. 5.**
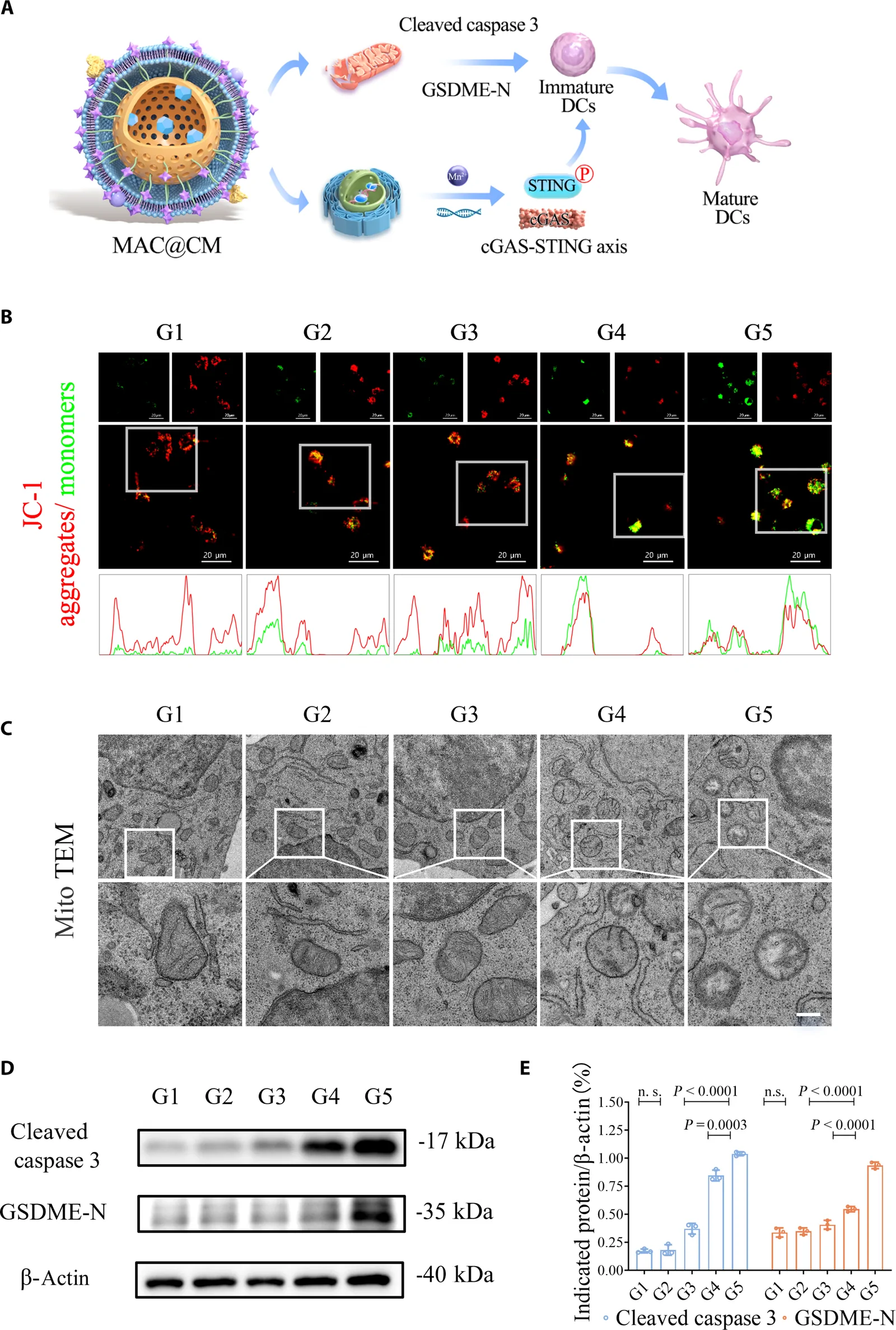
In vitro evaluation of the synergistic antitumor effects of MAC@CM. (A) Diagram of the process of dendritic cell (DC) maturation induced by MAC@CM under laser irradiation. (B) Observations of mitochondrial membrane potential in K7M2 cells following JC-1 dye staining. Scale bar: 10 μm. (C) The transmission electron microscope (TEM) images of K7M2 cells after different treatments. (D and E) The expression of the pyroptosis-associated proteins (i.e., GSDME-N and cleaved caspase 3) in K7M2 cells was analyzed using western blotting. Scale bar: 20 nm. The laser (660 nm) power density and irradiation time were 1.20 W/cm^2^ and 5 min, respectively. G1: Control + L, G2: M@CM + L, G3: MA@CM + L, G4: MC@CM + L, and G5: MAC@CM + L. Data in (E) is reported as the mean value ± SD, *n* = 3. The statistical significance was obtained using a 1-way analysis of variance (ANOVA).

As shown in Fig. 5A, the effective formation of a tumor intracellular ROS storm in OS damages multiple intracellular components, including the nucleus, mitochondria, and multiple organelles. These lesions lead to activation of the caspase 3 pathway, which subsequently cleaves the Gasdermin E (GSDME) and releases its N-terminal fragment. The released fragments subsequently oligomerize to form cell membrane pores, which facilitate the efficient release of DAMPs and dsDNA from OS cells. To verify the process, the JC-1 assay was first used to evaluate the effect of MAC@CM on mitochondrial membrane potential. The shift of JC-1 fluorescence from red to green indicated the loss of membrane potential and mitochondrial dysfunction. As shown in Fig. 5B, the cells in Control group presented predominantly red fluorescence, whereas cells treated with MAC@CM combined with laser irradiation exhibited intense green fluorescence. This group showed the brightest green signal in all conditions, reflecting the most severe mitochondrial dysfunction, attributed to increased ROS production by Ce6 in synergy with CA.

Then, we used TEM to observe the damage of mitochondria in cells. As shown in Fig. 5C, mitochondria in the MAC@CM group showed severe vacuolation and swelling, with complete disappearance of mitochondrial cristae, indicating the most severely injured. This finding is consistent with the JC-1 results, confirming that ROS play a damaging role in mitochondria. In order to explore the activation of cleaved caspase 3 in the cytoplasm and the effective cleavage of GSDME, we used western blotting to verify the changes of this pathway. Compared with the other groups, the expression levels of cleaved caspase 3 and GSDME-N in the MAC@CM + L group were substantially higher after laser irradiation than the other groups (Fig. 5D and E), indicating that cleaved caspase 3 was substantially activated after laser irradiation, and GSDME was successfully cleaved to GSDME-N, which provided favorable conditions for the formation of plasma membrane pores.

### Evaluation of ICD and cGAS signaling pathways during DC maturation in vitro

**Fig. 6.**
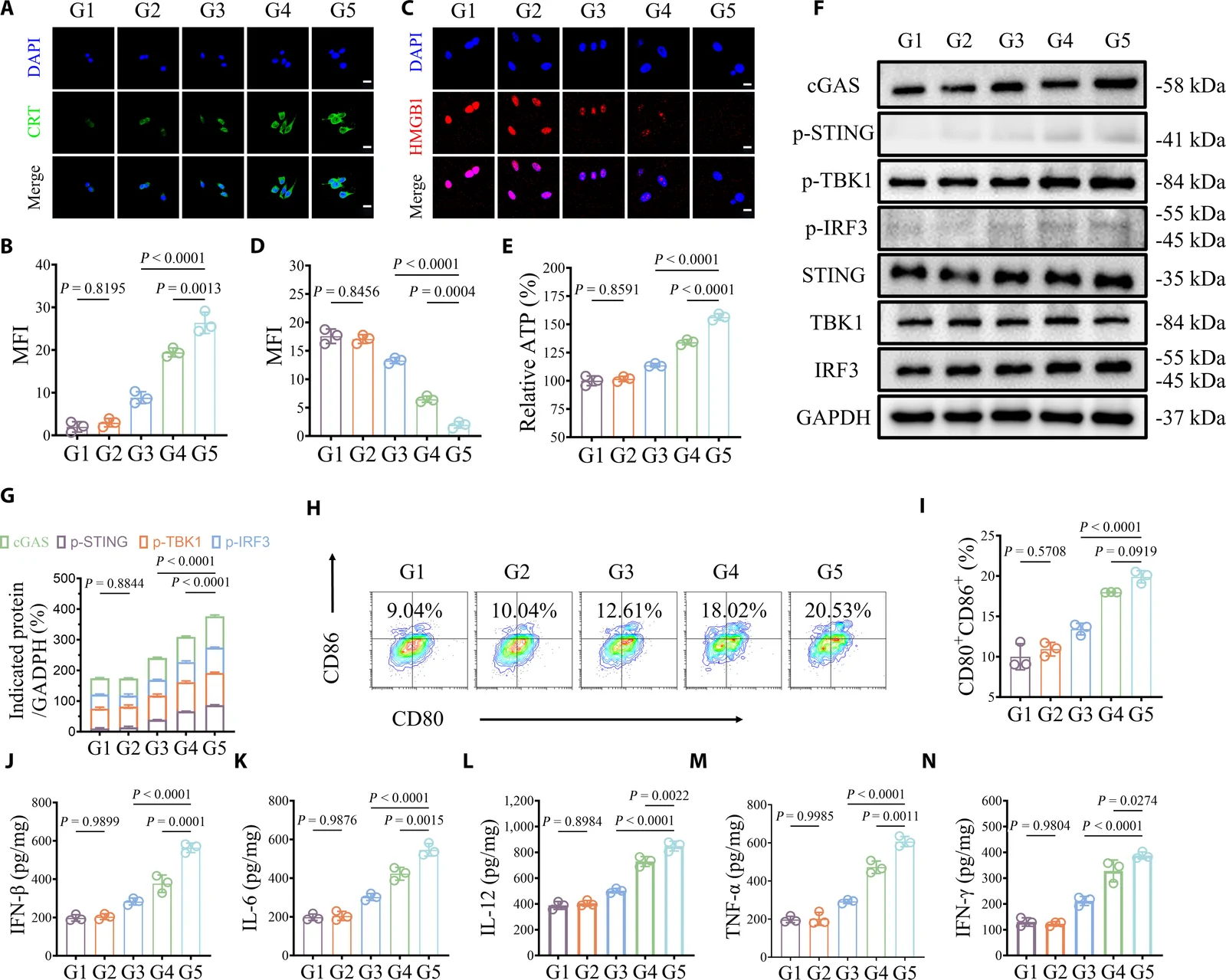
In vitro immunogenic cell death (ICD) evaluation and dendritic cell (DC) maturation analysis. (A and C) Representative distributions of calreticulin (CRT) and high mobility group box 1 protein (HMGB1) on K7M2 cells after various treatments. Scale bar: 10 μm. (B and D) The relative fluorescence intensities of the CRT and HMGB1 signals analyzed using ImageJ. (E) Adenosine triphosphate (ATP) levels in cell supernatants. (F and G) The expression of the cyclic guanosine monophosphate-adenosine monophosphate synthase–stimulator of interferon genes (cGAS-STING) pathway proteins (i.e., -/p-STING, -/p-TBK-1, and -/p-IRF-3) in DCs was analyzed using western blotting (WB). (H) The percentages of mature DCs and (I) rate of CD80^+^CD86^+^ in CD11c^+^CD45^+^ cells following coculture with K7M2 cells that had been pretreated in different ways. The levels of cytokines (J) interferon-β (IFN-β), (K) interleukin-6 (IL-6), (L) IL-12, (M) tumor necrosis factor-α (TNF-α), and (N) IFN-γ in the K7M2 and DCs cocultured cells were determined using enzyme-linked immunosorbent assay. The laser (660 nm) power density and irradiation time were 1.20 W/cm^2^ and 5 min, respectively. G1: Control + L, G2: M@CM + L, G3: MA@CM + L, G4: MC@CM + L, and G5: MAC@CM + L. Data in (B), (D), (E), (G), (I), and (J) to (N) are reported as the mean values ± SD, *n* = 3. The statistical significance was obtained using a 1-way analysis of variance (ANOVA).

After verifying caspase-3-dependent GSDME cleavage and membrane pore formation, this study evaluated key ICD-related events: CRT exposure on the cell surface, high mobility group box 1 protein (HMGB1) release from the nucleus, and extracellular adenosine triphosphate secretion. These markers indicate that ICD status is substantially enhanced, and their functions in activating the cGAS-STING pathway and promoting DC maturation can be elucidated by analyzing the mechanism of action. The experiments showed that laser irradiation had little effect on CRT exposure. The cells exposed to CRT increased slightly in the MA@CM group but more substantially in the MC@CM group. Notably, cells in the MAC@CM group exhibited the highest CRT exposure (Fig. 6A and B), which is attributed to the role of CA further enhancing the favorable environment for PDT. Furthermore, compared to the other groups, the cells treated with MAC@CM + L released higher levels of HMGB1 and adenosine triphosphate content (Fig. 6C to E). In conclusion, our findings indicate that MAC@CM combined with laser irradiation is an effective method to enhance the release of DAMPs by inducing an enhanced pyroptosis-ICD.

The activation of the cGAS-STING pathway plays a powerful role in inducing innate immunity [30–33]. Due to the effects of the ROS storm on OS cells, coupled with the pore-forming activity of the GSDME-N fragment at the plasma membrane, a large amount of dsDNA is released from OS cells through pores in the cell membrane and subsequently taken up by antigen-presenting cells (APCs), such as DCs. The dsDNA that enters the DCs is captured by cGAS protein, while Mn^2+^, which is degraded by the TME, can also enter the DCs, further enhancing the sensitivity of cGAS to capture dsDNA and delivering it to the STING protein located in the endoplasmic reticulum, leading to its phosphorylation [34,35]. This, in turn, causes the phosphorylation of downstream TANK-binding kinase 1 and interferon regulatory factor 3, resulting in the release of interferon-β and activation of innate immunity. To verify this process, we cocultured tumor cells from different experimental groups with DC cells. After 36 h, we performed western blot analysis on the cells, and the results are shown in Fig. 6F and G. There is a substantial increase in cGAS-STING-related proteins compared with Control + L group.

To further evaluate the effect of the combined immunotherapy strategy based on adaptive immunity and innate immunity on the maturation of DCs, cells from coculture systems were collected, immunostained, and subjected to flow cytometric analysis for mature DC biomarkers (CD45^+^CD11c^+^CD80^+^CD86^+^, gating strategy in Fig. S14). The results demonstrated that Ce6 pretreatment increased the proportion of mature DCs beyond the level achieved by laser irradiation alone (9.04%) (Fig. 6H and I). In particular, the highest DC maturation rate (20.53%) was observed in the MAC@CM + L pretreatment group. To further validate the role of Mn^2+^ in this therapeutic strategy, we introduced sodium dimercaptopropanesulfonate (Na-DMPS) to inhibit intracellular Mn^2+^ concentration. The tumor cells were subsequently treated as follows: Control + L, MAC@CM + L, MAC@CM + Na-DMPS + L, and the treated cells were cocultured with DCs. The cells were analyzed by FCM. Compared with MAC@CM + L group, the ratio of mature DCs in Na-DMPS group was substantially reduced (Fig. S15), indicating that Mn^2+^ had a sensitizing effect on the activation of cGAS-STING pathway in DCs during this process. Finally, we collected the supernatant of cocultured cells and detected the levels of related inflammatory factors and type I interferons by enzyme-linked immunosorbent assay. As shown in Fig. 6J to N, the results showed that MAC@CM has substantial proinflammatory effect, which was consistent with the above experimental data. Overall, this synergistic strategy of enhancing pyroptosis-driven adaptive immunity and activating the cGAS-STING innate immune pathway provides a promising immunotherapeutic option for cancer treatment.

### RNA sequencing analysis of K7M2 and DC cocultured cells treated with Control + L, MAC@CM+L

**Fig. 7.**
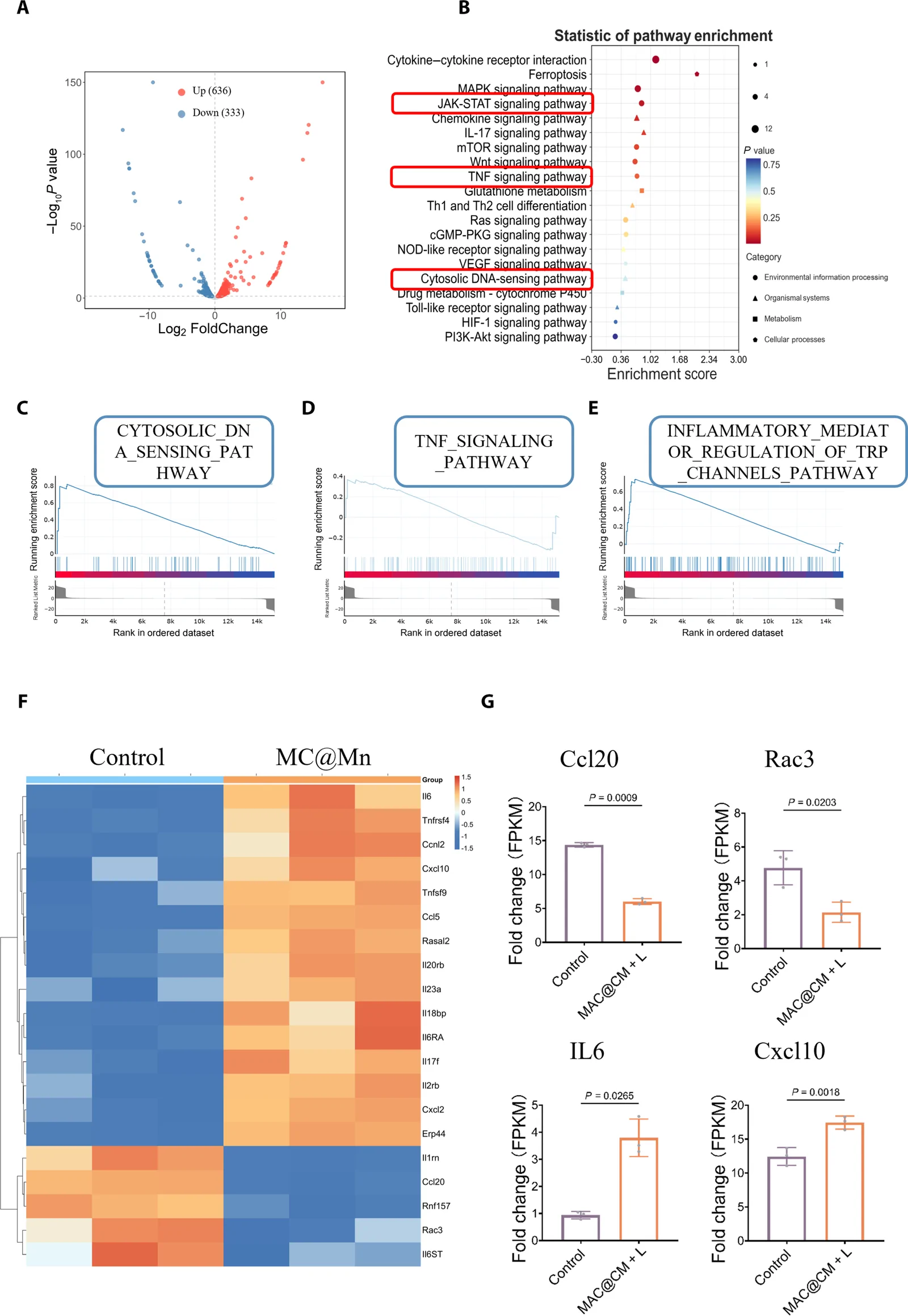
In vitro RNA sequencing analysis. (A) Volcano plot of differentially expressed genes. (B) Dot plot depicting the Kyoto Encyclopedia of Genes and Genomes (KEGG) pathways of differentially expressed genes in K7M2 and dendritic cell (DC) cocultured cells. (C to E) The Gene Set Enrichment Analysis (GSEA) related with immunoreaction and antigen presentation in cocultured cells. (F) Heatmap of gene expressions related with antigen presentation and immunoreaction in cocultured cells with different treatments. (G) Relative expression (Fragments Per Kilobase of exon model per Million mapped fragments [FPKM]) of differentially expressed genes related with antigen presentation and immunoreaction. *n* = 3. Data in (G) are reported as the mean value ± SD, *n* = 3. The statistical significance was obtained using a 1-way analysis of variance (ANOVA).

To further explore the mechanism of MAC@CM on the coculture system of K7M2 cells and DCs, we used high-throughput next-generation sequencing technology to sequence the mRNA of cocultured cells under different treatment conditions. A total of 11,944 genes were subjected to transcriptome analysis. First, we performed principal component analysis on the gene expression of cocultured cells under different treatment conditions and analyzed the gene expression of cocultured cells under different treatment conditions; the results showed that principal component 1, principal component 2, and principal component 3 explained 20%, 20%, and 20% of the total detectable gene expression, respectively (Fig. S16), indicating substantial differences in gene expression among the different treatment groups, and there were no outliers or highly similar samples within each treatment group. The gene expression differences between the different drug-treated groups were then illustrated by a volcano plot (Fig. 7A), which showed that compared with the control group, 636 genes were up-regulated and 333 genes were down-regulated in the MAC@CM + L-treated group.

Additionally, we compared these differentially expressed genes with the Kyoto Encyclopedia of Genes and Genomes database (Fig. 7B). The results indicated that cells treated with MAC@CM + L led to substantial changes in immune-related signaling pathways, including cytokine–receptor interactions, cytosolic DNA sensing pathways, Janus kinase-signal transducer and activator of transcription signaling pathways, tumor necrosis factor signaling pathways, nucleotide-binding oligomerization domain/Toll-like receptor signaling pathways, and antigen processing and presentation. Notably, the cytosolic DNA sensing pathways closely related to cGAS-STING activation, specifically pathways [34,35], were found to have stronger correlations after treatment with MAC@CM + L. In addition, using Gene Set Enrichment Analysis (Fig. 7C to E), after treatment with MAC@CM + L, pathways related to cytosolic DNA sensing closely associated with cGAS-STING activation, as well as tumor necrosis factor and inflammatory pathways that synergize after cGAS-STING pathway activation, were substantially enriched. These results further corroborate previous studies.

Finally, from the differentially expressed genes of cocultured cells under various treatments, 20 genes involved in tumor cell development, inflammatory factors, and induced immune responses were selected and represented in a heatmap (Fig. 7F). Compared to cells treated with control + L, genes related to the latter 2 processes were up-regulated in cells treated with MAC@CM + L. However, compared to MAC@CM + L, genes related to tumor cell development showed up-regulation in cells treated with control + L. Compared to cells treated with control + L, genes related to the latter 2 processes were up-regulated in cells treated with MAC@CM + L. However, compared to MAC@CM + L, genes related to tumor cell development showed up-regulation in cells treated with control + L. Next, select 4 genes of particular interest and quantify their gene expression (Fig. 7G). Compared to the cells treated with control + L, the expression of the tumor cell growth and migration-related genes Ccl20 and Rac3 is reduced in the cells treated with MAC@CM + L, while the expression of the inflammation and immune response-related genes Cxcl10 and Il6 is substantially increased, which aligns with the expected results previously demonstrated in vitro. In conclusion, transcriptomic analysis further validated the mechanism by which cells regulated by MAC@CM. The substantial increases in CXCL10 and interleukin-6 were consistent with the results expected from previous in vitro experiments [36]. Overall, transcriptomic analysis further confirms the mechanism by which MAC@CM regulates OS cells.

### In vivo antitumor efficiency of MAC@CM

Before evaluating the antitumor effect of MAC@CM in vivo, we first conducted a preliminary examination of its biosafety. Blood biochemical analysis after intravenous injection revealed that the levels of key indicators such as total protein (TP), albumin (Alb), alanine aminotransferase (Alt), aspartate transaminase (AST), blood urea nitrogen (Bun), uric acid (UA), total bile acid (TBA), and direct bilirubin (DBIL) were all comparable to Control group (Fig. S17). In addition, pathological analysis of tissues after laser irradiation by H&E staining and Masson trichrome staining showed that no obvious damage was observed compared to the nonirradiated control group (Fig. S18), suggesting that laser irradiation may be beneficial for the treatment of lung cancer; this confirmed the safety of the adopted laser parameters.

**Fig. 8.**
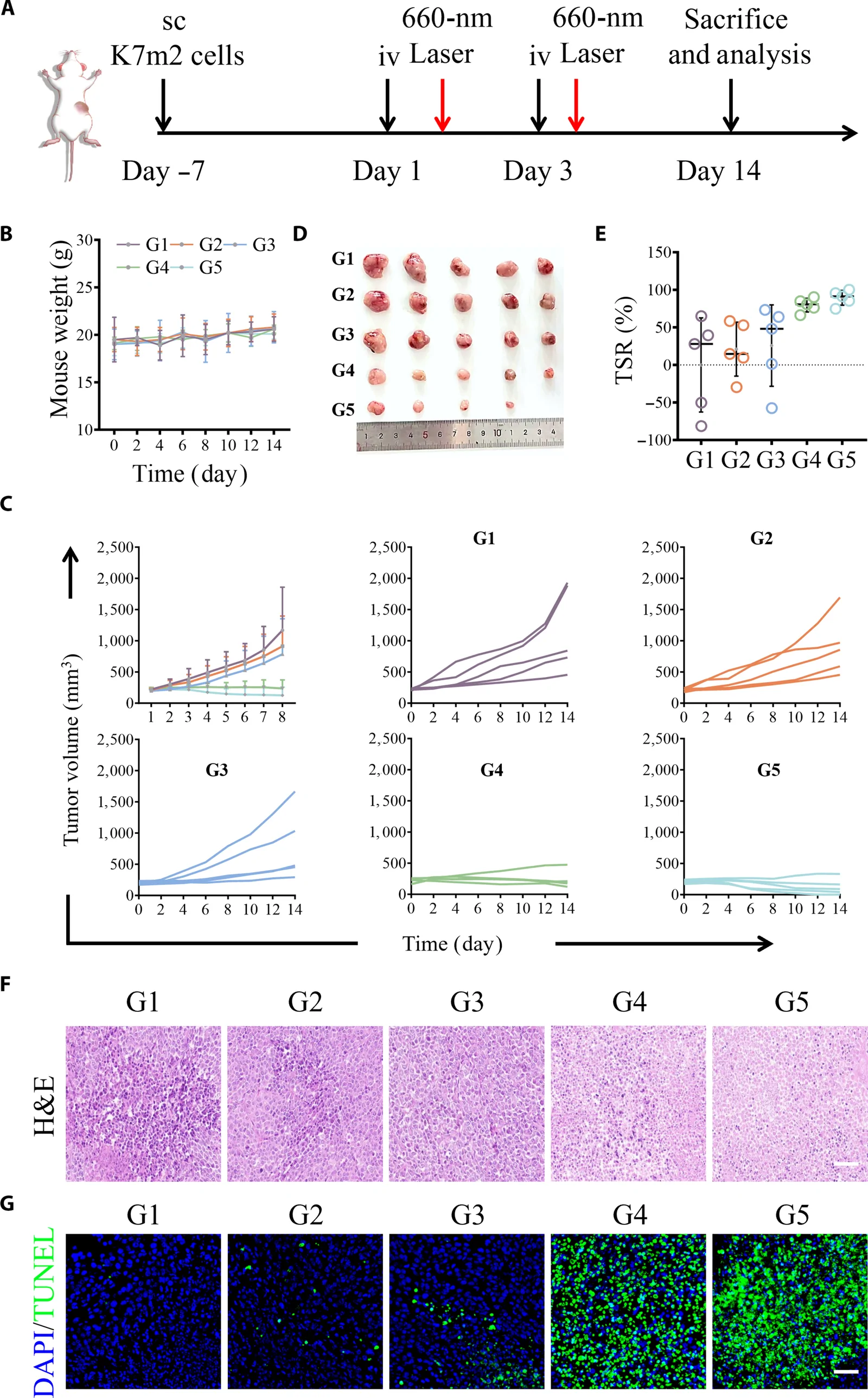
In vivo evaluation on the antitumor efficiency after different treatments. (A) Diagram illustrating the experimental protocol used to evaluate the antitumor efficiency of MAC@CM in vivo. (B) The mouse weight during 14 d. (C) Tumor growth curves during different treatments. (D) Tumor images from the various groups on the 14th day after treatment. (E) Tumor suppression rate (TSR) of different treatment on the last day during treatment. Representative images of tumor sections treated with (F) hematoxylin and eosin (H&E) staining and (G) TUNEL (terminal deoxynucleotidyl transferase dUTP nick end labeling) following various treatments. Scale bar: 100 μm. The laser (660 nm) power density and irradiation time were 1.20 W/cm^2^ and 5 min, respectively. G1: Control + L, G2: M@CM + L, G3: MA@CM + L, G4: MC@CM + L, and G5: MAC@CM + L. Data in (B), (C), and (E) are reported as the mean values ± SD, *n* = 5. The statistical significance was obtained by using a 1-way analysis of variance (ANOVA). sc, subcutaneous; iv, intravenous.

Following confirmation of its tumor-targeting efficacy and systemic biosafety, we evaluated the antitumor potential of MAC@CM in K7M2 mice model. The mice were randomly divided into 5 groups (*n* = 5 per group): Control + L, M@CM + L, MA@CM + L, MC@CM + L, and MAC@CM + L, with the specific administration timeline shown in Fig. 8A. Mice in each group received laser irradiation (660 nm, 1.20 W/cm^2^, 5 min) after intravenous injection of the corresponding preparation for 12 h. Throughout the 14-d study, body weight and tumor volume were monitored every 2 d. No substantial body weight loss was observed in any groups (Fig. 8B). In addition, H&E-stained sections of major organs (heart, liver, spleen, lung, and kidney) showed no substantial pathological changes (Fig. S19), collectively indicating that the MAC@CM + L regimen was well-tolerated at the administered dose. The curves of tumor volume change were recorded to show the tumor suppression effect of each group (Fig. 8C). The control + L group showed the least treatment effect on tumors, while the MA@CM + L group suppressed part of the tumor growth rate in the initial stage, but the effect was not substantial. The MC@CM + L group exhibited a more pronounced tumor growth inhibition, and subsequently, the MAC@CM + L group had the slowest tumor volume growth rate. This suggests that the combination strategy of CA and Ce6 is superior to monotherapy in terms of tumor ablation efficacy. At the end point, all tumors were resected, photographed, and histologically evaluated. Ex vivo analysis revealed that the tumor volume of the MAC@CM + L group was substantially smaller than the other groups (Fig. 8D). Consistent with this, tumor volume measurements confirmed that this group achieved the highest tumor ablation rates (Fig. 8E). The presence of extensive cell death within the tumors of the MAC@CM + L group was further confirmed by histopathology assessment with H&E and TUNEL staining (Fig. 8F and G). These in vivo findings are consistent with the potent ablative effects previously observed in vitro.

### Systemic antitumor immune response activation in vivo via MAC@CM

**Fig. 9.**
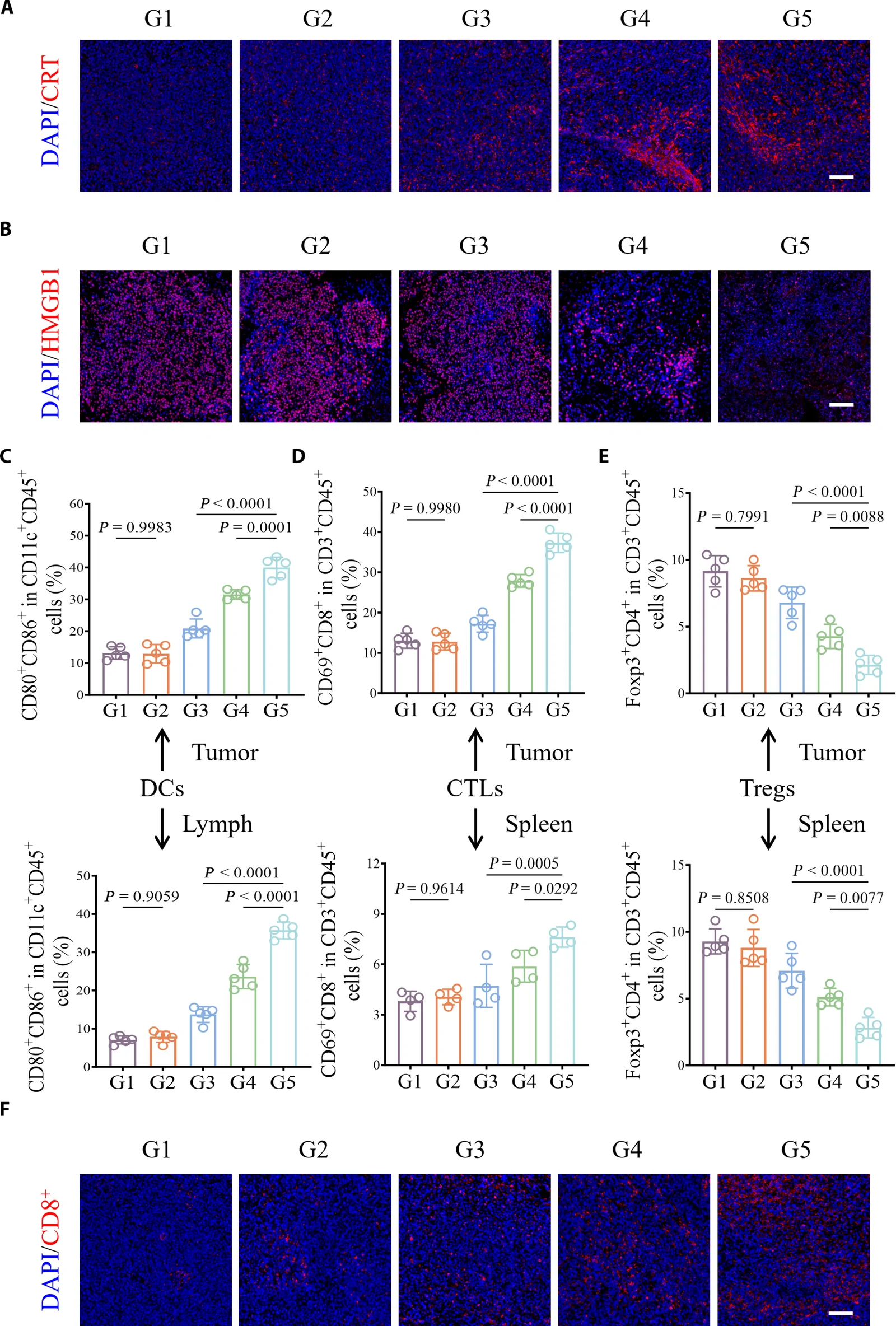
In vivo evaluation on the immune response after different treatments. The distributions of (A) calreticulin (CRT) and (B) high mobility group box 1 protein (HMGB1) in tumor sections were analyzed by an immunofluorescence assay. Scale bar: 100 μm. (C) The percentages of mature dendritic cells (DCs) in lymph nodes and tumors (*n* = 5). The percentages of (D) cytotoxic T lymphocytes (CTLs) and (E) regulatory T cells (Tregs) in tumors and spleens (*n* = 4). (F) The distributions of CD8^+^ in tumor sections were analyzed by an immunofluorescence assay. Scale bar: 100 μm. The laser (660 nm) power density and irradiation time were 1.20 W/cm^2^ and 5 min, respectively. Data in (C) are reported as the mean value ± SD, *n* = 5. G1: Control + L, G2: M@CM + L, G3: MA@CM + L, G4: MC@CM + L, and G5: MAC@CM + L. The statistical significance was obtained by using a 1-way analysis of variance (ANOVA).

The heterogeneity of tumor immune microenvironment is a key factor in tumorigenesis, progression, and treatment failure [37,38]. In this study, MAC@CM + L treatment was shown to substantially promote pyroptosis ICD and activate APCs and cGAS-STING innate immune pathways. To explore the translational significance of this mechanism, we detected CRT and HMGB1 in tumor sections by CLSM and then evaluated the changes of corresponding biomarkers in vivo. At the beginning, tumor tissues were stained, using fluorescently labeled CRT and HMGB1 antibodies, and subjected to CLSM. MAC@CM + L treatment substantially increased the expression levels of CRT and HMGB1 in tumor tissues (Fig. 9A and B and Fig. S20), indicating its efficient ICD function. Then, we detected the immune cells in the sentinel lymph node, spleen, and tumor to explore the activation of the typical antitumor immune response and the improvement of the tumor immune microenvironment. The gating strategy for FCM analysis is shown in Fig. S21. DC maturation is the key step by which APCs prime naive T cells and is important for initiating and maintaining antitumor immunity. We evaluated DC maturation in tumors and draining lymph nodes after the different treatments by FCM. The MAC@CM + L group had the highest percentage of mature DCs in lymph nodes (34.45%, 6.85%) and tumors (35.7%, 2.48%) than the other groups (Fig. 9C and Fig. S22A and B), indicating that this treatment promotes DC maturation and stimulates immune response.

Because CTLs (CD45^+^ CD3^+^ CD8 ^+^ CD69^+^) are directly involved in the killing of tumor cells and Tregs (CD45^+^ CD3^+^CD4^+^Foxp3^+^) are immunosuppressive in the TME, we measured the proportion of CTLs in tumors and spleens to assess the tumor immune microenvironment. FCM analysis revealed that the MAC@CM + L group had more CTLs (tumors: 31.70% ± 3.84%; spleens: 8.31 ± 1.23%) and fewer Tregs than the other groups (Fig. 9D and E and Fig. S22C), suggesting that the MAC@CM + L group has more Tregs than the other groups; this means that the computed CTLs/Tregs ratio was substantially higher in this group (Fig. S22D), indicating a positive shift toward immune activation rather than suppression. The histological analysis of infiltrating tumor CD8^+^ T cells (Fig. 9F), CTLs, and Tregs is consistent with the FCM analysis results (Fig. S23). We found that MAC@CM + L activated the antitumor immune response and reprogramming immunosuppressive TME due to previous destruction of tumor hypoxic microenvironment and antioxidant protection system by HMnO_2_ and CA, provided the foundation for the ROS storm caused by Ce6 with laser irradiation, enhanced the immune synergistic action of pyroptosis-ICD and cGAS-STING pathway activation, and effectively adaptive and innate immune activation against tumors.

In conclusion, MAC@CM + L enables effective immune-mediated tumor suppression by synergistically enhancing ICD and reprogramming the TME to establish a favorable immune environment.

### Distant tumor suppression in vivo

Efficient activation of systemic antitumor immune responses is essential to prevent recurrence and metastasis [39,40]. Prompted by the immunostimulatory outcomes observed in previous assessments, a bilateral K7M2 tumor model, bearing both a primary (treated) and a distant (untreated) tumor, was established to evaluate whether the immune synergy mediated by MAC@CM + L could exert a systemic therapeutic effect against distant, nonirradiated site.

**Fig. 10.**
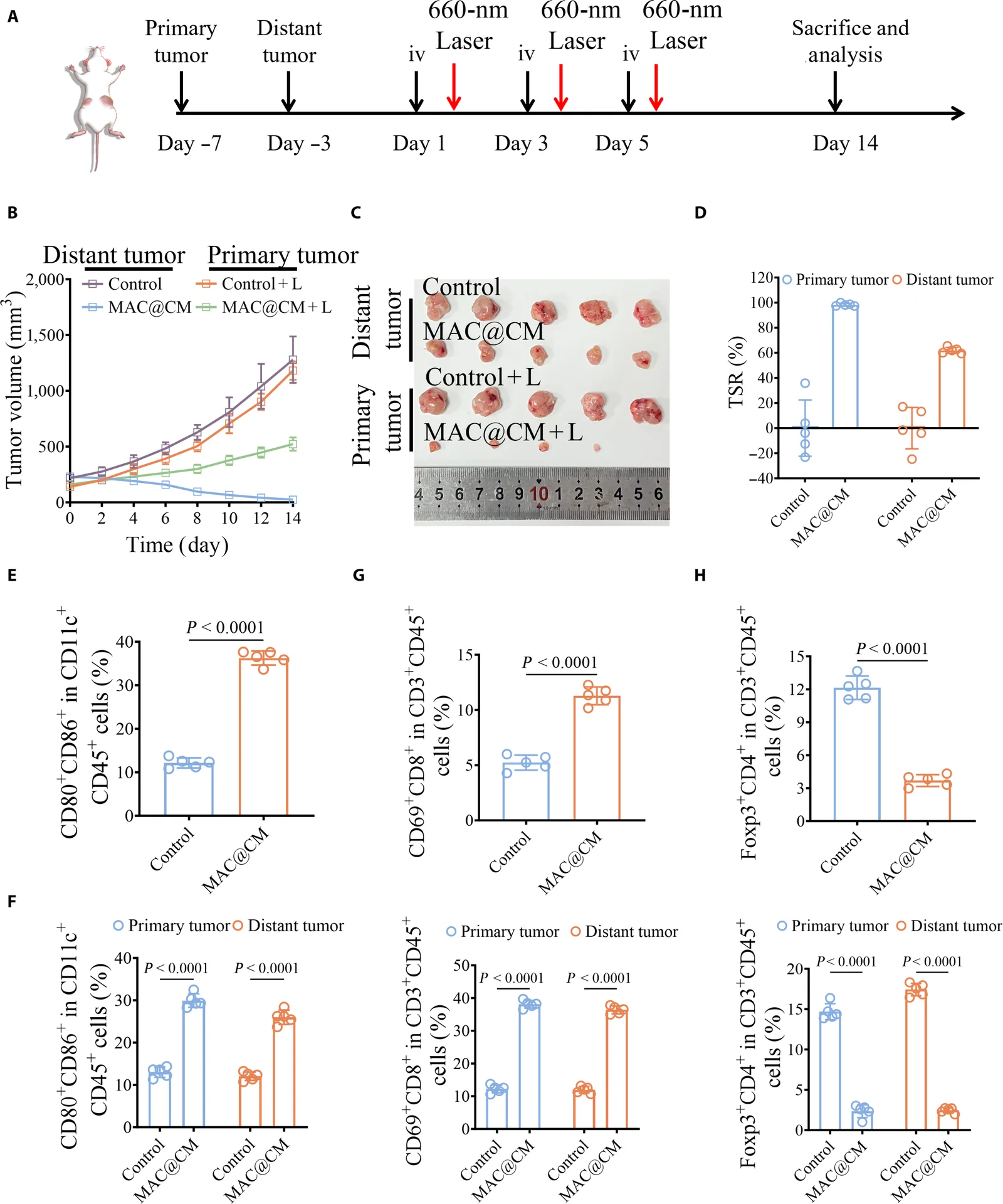
In vivo evaluation on the systemic immune response following different treatments. (A) Diagram illustration of the treatment schedule of MAC@CM against primary and distant tumors. (B and C) Tumor growth curves of mice. (D)Tumor images from the various groups on the 14th posttreatment day. (E and F) The percentages of mature dendritic cells (DCs) in lymph nodes and tumors. The percentages of (G) CTLs and (H) regulatory T cells (Tregs) in tumors and spleens. G1: Control + L, G2: M@CM + L, G3: MA@CM + L, G4: MC@CM + L, and G5: MAC@CM + L. Data in (A), (D), and (E) to (H) are reported as the mean values ± SD, *n* = 5. The statistical significance was obtained using a Student *t* test. iv, intravenous.

A diagram of the bilateral tumor model establishment is presented in Fig. 10A. Briefly, K7M2 cells were implanted subcutaneously on the right flank to form the primary tumor. Distant tumor was established on the left flank 4 d later. Treatment commenced when the primary tumor volume reached 150 mm^3^. MAC@CM was delivered intravenously, followed 12 h later by irradiation of only the primary tumor (660-nm laser, 1.20 W/cm^2^, 5 min) after each injection. Tumor volumes at both sites were recorded every 2 d throughout the period. On the 14th day after the treatment, FCM imaging and analysis were carried out on primary and distant tumors. Compared to the Control + L group, both primary and distant tumors were slower growing in the MAC@CM + L group (Fig. 10B and C). This implies that treatment with MAC@CM + L inhibited the growth of the primary tumor but also suppressed the distant untreated tumor.

Photographic images of excised tumors confirmed this growth inhibition trend (Fig. 10D) but no substantial body weight difference between groups (Fig. S24). In order to summarize the systemic immune response, immune cells were measured both in primary tumors and distant tumors. Compared with Control, MAC@CM + L showed a higher ratio of mature DCs and CTLs (Fig. 10E to G and Fig. S25A and B) and markedly lower Tregs (Fig. 10H and Fig. S25C). The consequent increase in the CTLs/Tregs ratio further corroborated these shifts (Fig. S25D and E). Collectively, these data demonstrated that MAC@CM + L treatment effectively reversed the immunosuppressive TME and instigates a potent systemic antitumor immune response. In summary, this combined immunotherapeutic strategy, which activated both adaptive and innate immunity via MAC@CM, potently stimulated systemic antitumor immunity, thereby holding promise for suppressing recurrence and metastasis.

### Study limitations

This study has several limitations. First, in vivo treatment evaluation was limited to a 14-d observation period to enable simultaneous collection of tumor tissues and immune-related organs for histological and immunological analysis. Therefore, long-term tumor control, recurrence, and overall survival have not been fully evaluated. Although body weight monitoring, serum biochemical analysis, and organ H&E staining in mice indicate favorable short-term biosafety, an extended observation period is needed to further evaluate the treatment durability, chronic toxicity, and efficacy of MAC@CM + L, pharmacokinetics, biodistribution, metabolic clearance, and potential long-term retention. Second, current Ce6-based PDT systems rely on irradiation at 660 nm. Although this wavelength matches the absorption properties of Ce6 and is applicable to the subcutaneous tumor model used in this study, its limited tissue penetration may limit its application in deep osteosarcoma lesions. Future studies may explore photosensitizers at longer wavelengths or photoresponsive systems with improved penetration depths. In addition, potential sex-related differences in the elimination and utility of osteosarcoma in situ still need to be further evaluated.

Finally, although MAC@CM + L was shown to induce mitochondrial damage, promote GSDME-mediated pyroptosis, activate ICD and cGAS-STING-related immune effects, and provide a novel mechanism of protection against pyroptosis; however, specific pathway inhibitors or gene silencing studies are still needed to confirm the causal relationship between these events.

## Conclusion

We have successfully prepared a novel hierarchically targeted nanomedicine system based on tumor cell membranes and CA modifications (MAC@CM), MAC@CM the nanoparticles that are endocytosed by OS cells first release the shell-modified CA, which efficiently lodges at the site of the OS through the tumor cell membrane, targeting damaged mitochondria and generating H_2_O_2_ synergized with HMnO_2_ to alleviate the hypoxic microenvironment of tumors, while HMnO_2_ and CA synergized to degrade GSH, disrupting the antioxidant defense mechanism of OS cells, and promoting the development of OS cells, the combination of the two provides a good environment for PDT. Finally, under the action of adjustable PDT, a ROS storm is generated, which further damages mitochondria and forms a mitochondrial-damage amplification effect to promote pyroptosis of tumor cells.

Pore formation by GSDME-N triggers the release of DAMPs and dsDNA, which, sensitized by Mn^2+^, potentiate cGAS-STING signaling in tumor-adjacent DCs. This cascade promotes DC maturation and subsequent T-cell infiltration, effectively reversing OS immunosuppression, reshaping the TME, and culminating in systemic antitumor immunity capable of controlling distal disease. Transcriptomics further confirmed the regulatory role of MAC@CM in immune response, inflammatory chemotaxis, and antitumor.

Overall, leveraging tumor cell membrane camouflage, the amplified mitochondrial damage from CA-enhanced PDT, and systemic immune reactivation, this self-synergistic ROS-generating platform collectively achieves selective antitumor efficacy with favorable biosafety, offering a referential strategy for precision cancer therapy.

## Data Availability

Data generated during the current study are available from the corresponding authors upon reasonable request.

## Supplementary Materials

Supplementary 1 Supplementary Methods Figs. S1 to S25 Graphical Abstract
