# Supplementary material for: Photodynamically Sensitized Mitochondrial-Damage Amplification Strategy Promotes Pyroptosis-Induced Immunotherapy of Osteosarcoma
Source: Biomater Res. 2026 Sep 9;30:0392. doi: 10.34133/bmr.0392 (PMC13554715; doi:10.34133/bmr.0392)
Supplement: Supplementary file 1 [file bmr.0392.f1.zip › Supporting information (clean version).docx]

**Photodynamically sensitized mitochondrial damage amplification strategy promotes pyroptosis-induced immunotherapy of osteosarcoma**

*Tao Ding^a,b#^, Tongyin Xiong^c#^, Xinzhong Xu^a,b#^, Qiuting Sun^d^, Mengyu Liu^d^, Zhennan Fang^a,b^,Yang Li^a,b^, Zhiwei Wu^a,b^, Wenbiao Zhang^a,b^, Ping Zhou^d^, Hongyu Bao^c^, Xiaoyan He^c^, Chi Zhang^c*^, Dasheng Tian^a,b*^, Fei Huang^a,b*^.*

^a^ Department of Orthopedics, The Second Affiliated Hospital of Anhui Medical University, Hefei 230601, P. R. China

^b^ Institute of Orthopaedics, Research Center for Translational Medicine, The Second Affiliated Hospital of Anhui Medical University, Hefei 230601, P. R. China

^c^ Department of Orthopaedics, Anhui Public Health Clinical Center, Hefei, 230000, P. R. China

^d^ School of Life Sciences, Anhui Medical University, Hefei 230011, P. R. China

^*^ Corresponding authors: *Dasheng Tian* (Email: tiandasheng@ahmu.edu.cn.); *Xiaoyan He* (hexiaoyan@ahmu.edu.cn.); *Fei Huang* (Email: huangfei@ahmu.edu.cn.)

^#^ *T.D., C.Z., and X.X*. contributed equally to this work.

**Materials and methods**

**Materials**

Chlorin e 6 (Ce6) and cinnamaldehyde (CA) were purchased from Sigma-Aldrich，Tetraethyl orthosilicate (TEOS), ammonium hydroxide and potassium permanganate (KMnO_4_) were both purchased from Innochem (Beijing, China), cell counting kit-8 (cck-8), Calcein-AM/PI double staining kit, JC-1 Kit, ROS detection kit, H_2_O_2_ detection kit, superoxide detection Kit, enhanced ATP assay kit, bicinchoninic acid (BCA) protein quantification kit, were purchased from Beyotime (shanghai, China). Hoechst 33342 was obtained from Invitrogen. Recombinant murine granulocyte-macrophage colony-stimulating factor (GM-CSF) and recombinant murine interleukin-4 (IL-4) were purchased from PeproTech Inc. ELISA Kits (TNF-α, IFN-β and IL-6) were purchased from Shanghai Keshun Biotechnology Co., Ltd. Deoxyribonuclease I (DNase I) and collagenase IV were supplied by Yeasen Biotechnology (Shanghai) Co., Ltd. The primary antibody of β-actin (M20011F) and GAPDH (P60037S) was purchased from Abmart (Shanghai, China). The primary antibodies of high mobility group box-1(HMGB1, A19529, 1:100) were purchased from ABclonal (Wuhan, China). The primary antibody of cleaved caspase-3 (#9664, 1:1000) was obtained from Cell Signaling Technology Inc. The primary antibodies of anti-calreticulin (ab92516, 1:500) and anti-DFNA5/GSDME-N-terminal (ab215191, 1:1000) were supplied from Abcam. The primary antibodies of pSTING (72971S, 1:1000), STING (13647S, 1:1000), pTBK1 (5483S, 1:1000), TBK (3504S, 1:1000), pIRF3 (29047S, 1:1000), IRF3 (4302S, 1:1000) were supplied from Cell Signaling Technology. The primary antibodies of cGAS (26416-1-AP, 1:5000) was supplied from proteintech. The secondary antibodies of HRP goat anti-mouse IgG H&L (AS003, 1:8000) and HRP goat anti-rabbit IgG H&L (AS014, 1:8000) were supplied from ABclonal (Wuhan, China). All antibodies for flow cytometry (FCM) analysis, including APC/Fire 750 anti-mouse CD45 (Cat#103154, 1: 400), FITC anti-mouse CD11c (Cat#117306, 1: 400), PE anti-mouse CD80 (Cat#104708, 1: 200), Brilliant Violet 421 anti-mouse CD86 (Cat#105031, 1: 200), FITC anti-mouse CD3 (Cat#100204, 1: 300), PE anti-mouse CD4 (Cat#100408, 1: 200), PE/Cyanine7 anti-mouse CD8b.2 (Cat#140416, 1: 200), Brilliant Violet 421 anti-mouse FOXP3 (Cat#126419, 1: 100), Alexa Fluor 647 anti-mouse CD69 (Cat#104518, 1: 200), Brilliant Violet 421 anti-mouse/human CD11b (Cat#101236, 1: 200), and FITC anti-mouse Ly-6G/ Ly-6C(Gr-1) (Cat#108406, 1: 300) were supplied by Biolegend, Inc. All other reagents were obtained from commercial suppliers and used as received.

**Cells and culture conditions**

K7M2 cells were obtained from the China Center for Typical Culture Collection (Wuhan, China). The cells were cultured in DMEM supplemented with 10% fetal bovine serum (FBS) and 1% penicillin-streptomycin at 37℃ in a humidified 5% CO_2_ atmosphere. When the cells reached approximately 80% confluence, they were passaged by digestion with 0.25% trypsin–EDTA. The digestion was terminated by adding complete DMEM, and the cell suspension was centrifuged at 1000 rpm for 5 min. The collected cells were then resuspended in fresh complete medium and subcultured at a split ratio of 1:2.

**Animals**

Female Balb/c mice (6 weeks old) were bought from Jiangsu Jicui Yaokang Biological Technology Co., Ltd. (Nanjing, China). All animal experiments were carried out according to the relevant laws and guidelines of the Laboratory Animal Center of Anhui Medical University (approved number: LLSC20200329). K7M2-bearing mice were established by subcutaneously injecting the K7M2 cell suspension (approximately 5×10^6^ cells in 100 μL PBS) into the right hind legs of mice. Then stored at -80℃ for subsequent membrane derivation.

**Cell membrane derivation**

K7M2 cell membrane (CM) were obtained following a previously published protocol[23]. Briefly, frozen K7M2 cells were thawed and homogenized. The homogenized solution was centrifuged at 700 g for 10 min at 4℃ to remove the cell nuclei and intact cells. The supernatant was then centrifuged at 14,000 rpm for 30 min at 4℃ to remove the mitochondria by discarding the precipitation. After the supernatant was centrifuged again at 14,000 rpm for 30 min at 4℃, the CM was obtained.

**Characterization of MAC@CM**

The size and zeta potential of nanoplatforms containing different drugs in ultrapure water (0.2mg/mL, HMnO_2_) were measured by a dynamic light scattering (DLS) and Zetasizer (Nano ZS, Malvern Instruments). The sizes of M@CM, MA@CM, MC@CM, MAC@CM in PBS buffer over 7 days was also examined by DLS. To characterize the prepared samples, the morphology of the nanoplatforms after drug loading was first observed by transmission electron microscopy (TEM, JEOL JEM-2100). Subsequently, the drug loading content (DLC) for each component, Ce6 and CA, was respectively determined through measurements on an Edinburgh DS5 UV-Vis spectrophotometer.

**Characterization of the cell membrane proteins**

To characterize their protein composition, CM and MAC@CM samples were prepared for SDS-PAGE analysis. Samples were first denatured in SDS loading buffer at 100 °C for 10 min. Proteins were separated on 10% SDS-polyacrylamide gel run at 100 V for 2 h. Electrophoresis followed by Coomassie Blue staining washed to remove background staining. Protein bands were visualized with Tanon 1600.

**Western blotting**

Briefly, the proteins of all samples were extracted using radioimmunoprecipitation (RIPA) (Contains total protease inhibitors, phosphatase inhibitors) lysis buffer on ice for 20 min and then centrifuged at 12,000 g for 20 min. Total protein concentrations in the collected samples were quantified by the bicinchoninic acid (BCA) assay, and then total protein extracts were mixed with loading buffer and subjected to SDS-polyacrylamide gel electrophoresis (PAGE). After electrophoresis, separated proteins were transferred onto PVDF membranes using standard wet transfer method and blocked at room temperature for 1 hour in Tris buffered saline containing Tween 20 (TBST) and 5% (w/v) BSA incubated with the respective primary antibodies in blocking buffer at 4 °C overnight. Following staining with a secondary antibody at ambient temperature for 2 h, bound primary antibodies were labeled with ECL chemiluminescent reagents and visualized using a chemiluminescence imaging analysis system (Tanon 5200, China).

**Hemolysis assay**

The hemolytic activity of different concentrations of MAC@CM was evaluated by detecting the amount of hemoglobin released by mouse blood cells. Briefly, 160 μL of red blood cell (8%) suspension was mixed with 1840 μL ultrapure water (served as a positive control), 1840 μL of NaCl (0.9%, which served as a negative control), and 1840 μL of different concentrations of MAC@CM solution, respectively. After being kept at 37 ℃ for 6 h, The percentage of hemolysis is calculated according to the following formula: Hemolysis rate (%) = (Asample-Anegative)/(Apositive-Anegative) × 100%. In this formula, Asample, Anegative, and Apositive represent the absorbance values measured at 570 nm for the suspension of the test material, the negative control (0.9% sodium chloride), and the positive control (ultrapure water), respectively.

**Biosecurity study**

6-Week-old female mice were randomly divided into four groups. To verify the biosecurity of MAC@CM, MAC@CM was injected through the tail vein at an Ce6 concentration of 4.5 mg/kg per mouse. After three days, blood samples were sampled from the eye socket of the mice to test various indexes, including aspartate aminotransferase (AST), alanine aminotransferase (ALT), albumin (ALB), total protein (TP), uric acid (UA), blood urea nitrogen(BUN), direct bilirubin (DBIL) and total bile acid (TBA) during the treatment.

Figure S1 (a) XPS element quantification of HMnO_2_/PAH. (b) Standard curves for CA.

Figure S2 UV–vis absorption spectra of HMnO_2_, CA, Ce6 and MAC@CM.

Figure S3 XRD analysis of HMnO_2_ and MAC@CM

Figure S4 The size, PDI and Zeta Potential analysis for HMnO_2_, MA, MAC and MAC@CM. Data are presented as the mean value ± SD, n = 3.

Figure S5 Stability of HMnO_2_, MA, MAC, and MAC@CM in PBS over 7 days. Data are presented as the mean value ± SD, n = 3.

Figure S6 The (a) hemolysis picture and (b) ratio induced by MAC@CM with different drug concentrations. Data are presented as the mean value ± SD, n = 3.

Figure S7 Detection of MAC@CM Oxidative Damage Capacity. (a) different components of MAC@CM for GSH consumption capacity, (b) TMB experiment real photos. And (c) detection of singlet oxygen generation capacity of MAC@CM under different laser irradiation times.

Figure S8 (a) CLSM images and (b and c) FCM analysis of the cellular uptake of MAC@CM in K7M2 cells after incubating for different times. Scale bar: 10 μm. Data in (c) are presented as the mean value ± SD, n = 3. The statistical significance was obtained using a one-way ANOVA.

Figure S9 The fluorescence colocalization between lysosomes (red) and MAC@CM (Green) in K7M2 cells under different time treatments was detected by CLSM.

Figure S10 (a-d) The K7M2 cells viability after different treatments. (e) Cytotoxicity assay of different components at therapeutic concentrations on HUVEC cells. Data are presented as the mean value ± SD, n = 3. The statistical significance was obtained using a one-way ANOVA.

Figure S11 Combination index analysis of MAC@CM based on the Chou-Talalay method. (a) The curve of Dose-Fa. (b) The curve of Fa-CI.

Figure S12 Intracellular antioxidant system in K7M2 cells was disrupted by MAC@CM. The effects of different components in MAC@CM on (a) GSH, (b) H_2_O_2_, (c) superoxide, and (d) cell viability. Data in (a), (b) and (c) are presented as the mean value ± SD, n = 3.Data in (d) are presented as the mean value ± SD, n = 6. The statistical significance was obtained using a one-way ANOVA.

Figure S13 Healing assay was used to detect the migration of K7M2 cells in different treatment groups at different time.

Figure S14 Gating strategy to evaluate mature DCs (CD11c^+^CD80^+^CD86^+^) from the in vitro binary co-incubation system.

Figure S15 DCs matured during different experimental groups after Mn^2+^ inhibition treatment. The statistical significance was obtained using a one-way ANOVA.

Figure S16 The principal components analysis (PCA) between Control+L and MAC@CM+L

Figure S17 Blood biochemistry analysis of K7M2 tumor-bearing mice after injection of MAC@CM. Data are presented as the mean value ± SD, n = 3. The statistical significance was obtained using a one-way ANOVA (ns: no significance).

Figure S18 Representative photographs and histology of (a) mouse skin after treatment with different laser (660 nm) power densities for 5 min. Scale bars in H&E and Masson staining images: 50 μm.

Figure S19 H&E staining pictures of the major organs after treatment with MAC@CM. Scale bar: 50 μm.

Figure S20 Statistical analysis of mean immunofluorescence intensity of CRT and HMGB1. Data are presented as the mean value ± SD, n = 3. The statistical significance was obtained using a one-way ANOVA.

Figure S21 Gating strategy to sort mature DCs (CD11c^+^CD80^+^CD86^+^) from (a) lymph nodes and (b) tumors of K7m2-bearing Balb/c mice. Gating strategy to sort CTLs (CD45^+^CD3^+^CD8^+^CD69^+^), and Tregs (CD45^+^CD3^+^CD4^+^FOXP3^+^) from (c) spleens and (d) tumors of K7M2-bearing Balb/c mice.

Figure S22 (a) The representative FCM analysis of the percentages of mature DCs in

lymph nodes and tumors, (b) CTLs in tumors and spleens, (c) Tregs in tumors and

spleens. (d) CTLs/Tregs during spleen and tumor. Data are presented as the mean value ± SD, n = 5. The statistical significance was obtained using a one-way ANOVA.

Figure S23 The levels of CD69^+^ (a), and Foxp3^+^ (b) in tumor sections were examined by immunofluorescence at the end of the observation period. Scale bar: 50 μm.

Figure S24 Body weight changes of the K7M2 bilateral tumor model mice within 14

days. Data are presented as the mean value ± SD, n = 5

Figure S25 (a) The representative FCM analysis of the percentages of mature DCs in primary tumors, distant tumors and lymph nodes, (b) CTLs in primary tumors, distant tumors and spleens, (c) Tregs in primary tumors, distant tumors and spleens. CTLs/Tregs during (d) spleen and (e) tumor. Data are presented as the mean value ± SD, n = 5. The statistical significance was obtained using a student’s T-test and two-way ANOVA.
